# IPTG- and estradiol-inducible gene expression systems in the unicellular red alga *Cyanidioschyzon merolae*

**DOI:** 10.1093/plphys/kiaf575

**Published:** 2025-11-10

**Authors:** Takayuki Fujiwara, Shunsuke Hirooka, Shota Yamashita, Shin-ya Miyagishima

**Affiliations:** Department of Gene Function and Phenomics, National Institute of Genetics, Mishima, Shizuoka 411-8540, Japan; Genetics Program, Graduate University for Advanced Studies (SOKENDAI), Mishima, Shizuoka 411-8540, Japan; Department of Gene Function and Phenomics, National Institute of Genetics, Mishima, Shizuoka 411-8540, Japan; Department of Gene Function and Phenomics, National Institute of Genetics, Mishima, Shizuoka 411-8540, Japan; Department of Gene Function and Phenomics, National Institute of Genetics, Mishima, Shizuoka 411-8540, Japan; Genetics Program, Graduate University for Advanced Studies (SOKENDAI), Mishima, Shizuoka 411-8540, Japan

## Abstract

The genetically tractable unicellular red alga *Cyanidioschyzon merolae* has a remarkably simple genome (4,775 nucleus-encoded proteins) and cellular architecture. It contains only a single set of most membranous organelles, making it a valuable tool for elucidating the fundamental mechanisms of photosynthetic eukaryotes. However, as in other genetically tractable eukaryotic algae, previously developed systems for inducible gene expression rely on environmental stimuli such as heat shock or ammonium depletion, which impact cellular physiology and thus limit their usage. To overcome this issue, we developed IPTG- and estradiol-inducible gene expression systems in *C. merolae* in which the addition of these chemicals itself has no impact on cellular growth or the transcriptome. Additionally, we established IPTG- and estradiol-inducible protein knockdown systems and successfully degraded the endogenous chloroplast division protein DRP5B using the estradiol-inducible system. These systems facilitate functional genomic analyses in *C. merolae*, especially for understanding physiological mechanisms and their interactions in photosynthetic eukaryotes.

## Teaser

Controllable gene expression and protein levels in a red alga provide powerful tools for studying photosynthetic eukaryotes.

## Introduction

Many species of unicellular eukaryotic algae possess relatively simple intracellular architecture and compact genomes compared to land plant cells. They are cultured in media with a fully defined chemical composition, where vegetative cells of a single type, without the tissue differentiation seen in plants, are exposed to uniform environmental conditions such as light intensity, pH, temperature, and nutrient availability. Due to these characteristics, unicellular eukaryotic algae have the potential to serve as promising models for studying fundamental processes shared among photosynthetic eukaryotes (Miyagishima and Tanaka 2021).

Among various unicellular eukaryotic algae, the unicellular green alga *Chlamydomonas reinhardtii* has been widely used as a model system, with genetic manipulations becoming feasible over 30 yr ago (Goodenough 2023). Recently, procedures for genetic modification have also been developed for some other lineages of unicellular eukaryotic algae, including red algae, diatoms, coccolithophorids, and chlorarachniophytes (Apt et al. 1996; Hirakawa et al. 2008; Endo et al. 2016; Miyagishima and Tanaka 2021). However, genetic manipulation techniques for these algae are still not as well developed as those for other eukaryotic model systems.

The genetically tractable unicellular alga *Cyanidioschyzon merolae* exhibits an exceptionally simple cellular and genomic structure: the cell contains a single nucleus, mitochondrion, chloroplast, and peroxisome, as well as a single-layered endoplasmic reticulum and Golgi apparatus per cell (Kuroiwa et al. 2017). Its nuclear genome (16.5 mb) encodes only 4,775 proteins with low genetic redundancy (Matsuzaki et al. 2004; Nozaki et al. 2007). Because genetic manipulation of the *C. merolae* nuclear genome takes advantage of its high efficiency in homologous recombination, it can generate several types of transformants, such as gene knockouts and knock-ins, including the insertion of epitope or fluorescent tag-encoding sequences into gene loci (Imamura et al. 2009; Fujiwara et al. 2013a; Miyagishima and Tanaka 2021). In addition, 3 selectable markers for transformants, namely the uracil synthesis gene and chloramphenicol or blasticidin S resistance genes, are currently available (Imamura et al. 2009; Fujiwara et al. 2013a, 2017, 2021). These markers can be eliminated from transformants and reused for further genetic manipulation, allowing the editing of 2 or more loci (Takemura et al. 2018). Phylogenetically, *C. merolae* belongs to the class Cyanidiophyceae within Rhodophyta (red algae), which diverged from the algal ancestor of Viridiplantae (green algae and land plants) and Glaucophyta relatively soon after the chloroplast was integrated into the cell of the common ancestor of Archaeplastida via cyanobacterial endosymbiosis (Yoon et al. 2004; Reyes-Prieto et al. 2007). Among red algae, Cyanidiophyceae is the earliest-diverging group (Yoon et al. 2006). Furthermore, red algae gave rise to chloroplasts in various algal lineages, such as chrysophytes, dinoflagellates, cryptophytes, and haptophytes, through secondary endosymbiosis (Yoon et al. 2002; Keeling 2004; Sibbald and Archibald 2020; Strassert et al. 2021; Miyagishima 2023). Thus, due to its phylogenetic position, *C. merolae* plays a crucial role in comparative studies aimed at understanding the evolution of photosynthetic eukaryotes (Matsuzaki et al. 2004; Yoon et al. 2004; Misumi et al. 2005; Hirooka et al. 2022; Cho et al. 2023).

Owing to these unique characteristics, *C. merolae* has thus far facilitated a wide range of studies, including organelle biogenesis (Miyagishima et al. 1999, 2003; Nishida et al. 2003; Yagisawa et al. 2007, 2012, 2013 ; Yoshida et al. 2010; Imoto et al. 2017), the cell cycle (Kobayashi et al. 2011; Fujiwara et al. 2013b; Miyagishima et al. 2014; Yagisawa et al. 2020), nitrogen assimilation (Imamura et al. 2009), photosynthesis (Krupnik et al. 2013; Nikolova et al. 2017; Abram et al. 2020), epigenetics (Mikulski et al. 2017; Hisanaga et al. 2023), and RNA processing (Stark et al. 2015; Schärfen et al. 2022). These studies have been conducted through cytological, cytochemical, and reverse genetic analyses, often in combination with omics approaches. Additionally, research on the industrial applications of *C. merolae* has recently begun (Pancha et al. 2019; Izzo et al. 2021; Seger et al. 2023; Villegas-Valencia et al. 2023).

Since Cyanidiophyceae, including *C. merolae*, have undergone extensive genome reduction during evolution (Qiu et al. 2013; Cho et al. 2023), they have the advantage of encapsulating the minimal essential components required for functioning as a photosynthetic eukaryote. However, most of their genes are indispensable for survival and growth, making it unlikely to obtain knockout mutants. For phenotypic analysis of essential gene function inhibition, transient RNA interference (RNAi) treatment or a conditional expression system for the wild-type or dominant-negative form of the target gene can be effective. However, Cyanidiophyceae have lost key components of the RNAi machinery, such as Argonaute and Dicer (Casas-Mollano et al. 2008; Cerutti et al. 2011), making targeted RNA degradation unfeasible.

To overcome these limitations, heat shock-inducible gene expression and ammonium-dependent gene suppression systems utilizing endogenous promoters have been developed in *C. merolae* (Sumiya et al. 2014; Fujiwara et al. 2015). These systems have contributed to uncovering a retrograde connection between chloroplast division and mitotic progression (Sumiya et al. 2016) and identifying genes with cell cycle-dependent expression in *C. merolae* (Fujiwara et al. 2020). However, both heat stress (a temperature shift from 38 °C to 48 °C) and a change in the nitrogen source in the cultivation medium from nitrate to ammonium lead to significant metabolic and physiological alterations, as evidenced by substantial transcriptome changes (Kobayashi et al. 2014; Fujiwara et al. 2015). This is also the case in other eukaryotic algae, where inducible gene expression systems rely on temperature shifts or changes in the culture medium (Adler-Agnon et al. 2018; Kim et al. 2020; Shin et al. 2020). As an alternative approach, we recently developed a rapamycin-inducible protein knockdown system in *C. merolae* (Fujiwara et al. 2024). In this system, similar to other chemical dimerizer-induced protein knockdown systems used in yeasts and mammalian cells (Nishimura et al. 2009; Nabet et al. 2018; Tovell et al. 2019; Yesbolatova et al. 2020; Kanemaki 2022), rapamycin addition to the culture induces ubiquitination of the target protein by an E3 ligase, leading to its degradation by the proteasome within 2 to 3 h. However, this system still has practical limitations, such as the short duration of rapamycin's effect on target protein degradation (4 to 8 h) with a single administration and minor side effects on the cells (Fujiwara et al. 2024).

Under these circumstances, we focused on chemically inducible gene expression systems as an alternative approach that could be applied to *C. merolae.* To date, several reagent-inducible gene expression systems have been established in genetically tractable organisms, using reagents such as IPTG (a nonmetabolizable lactose analog), tetracycline (Tet, an inhibitor of bacterial and mitochondrial translation), dexamethasone (DEX, a synthetic glucocorticoid hormone), and estradiol (an estrogen steroid hormone). The Tet system has been widely used in animals and yeasts (Gossen and Bujard 1992; Ishii and Akiyoshi 2022), whereas the DEX and estradiol systems have been predominantly applied in land plants (Aoyama and Chua 1997; Zuo et al. 2000; Kubo et al. 2013). However, Tet, DEX, and estradiol exhibit some unintended side effects in these organisms (Amirsadeghi et al. 2007; Moullan et al. 2015; Upadhyay and Maier 2016). In contrast to these 3 reagents, IPTG demonstrates metabolic stability (Wyborski and Short 1991) and low cellular toxicity (toxicity > 50 mm in cultured mammalian cells, Figge et al. 1988). The IPTG system, which utilizes the *lacO*–LacI interaction, is widely used in bacteria due to its high inducibility of target gene expression. In this system, the activity of a promoter containing *lacO* operator sequences is regulated by the LacI repressor protein, which binds to *lacO* and inhibits gene transcription in the absence of IPTG. Upon IPTG addition, LacI dissociates from *lacO*, allowing RNA polymerase to initiate gene transcription. However, an efficient IPTG-based artificial on–off gene expression system has not yet been developed for eukaryotes, despite multiple efforts (Hu and Davidson 1987; Figge et al. 1988; Kjærulff and Nielsen 2015; Myung et al. 2020). In eukaryotic systems, issues such as leaky gene expression even in the absence of IPTG and low induction efficiency upon IPTG addition have been observed (Hu and Davidson 1987; Gossen and Bujard 1992).

By using the human interleukin-2 core promoter (*cIL2p*) with multiple *lacO* insertions, designed to be driven by a chimeric zinc finger–homeodomain 1 (ZFHD1) transcription factor, here we have developed a tightly regulated IPTG-inducible gene expression system in *C. merolae*. In addition, we have also developed an estradiol-inducible gene expression system in this alga, which sustains its effects for at least 72 h with a single dose. Moreover, by repurposing these systems, we have established protein knockdown systems. Importantly, IPTG and estradiol exhibit no detectable side effects on *C. merolae* cells. Together, these systems facilitate functional genomic analyses in *C. merolae*.

## Results

### Design and establishment of an IPTG-inducible gene expression system in *C. merolae* utilizing the ZFHD1-driven ZFHD1-binding sequences and the interleukin-*2* core promoter

In eukaryotic inducible gene expression analyses, the IPTG-inducible system was originally developed in cultured animal cells, but it was later gradually replaced by the Tet system because of issues such as leaky expression in the absence of IPTG and the relatively low efficiency of IPTG-mediated gene induction (Hu and Davidson 1987; Gossen and Bujard 1992). In such a situation, it was reported in *S. pombe* that the insertion of *lacO* immediately downstream of the TATA box in the *nmt* (no message in thiamine) promoter effectively repressed transcription initiation by LacI (Kjærulff and Nielsen 2015). Based on this finding, in this study, we aimed to develop an inducible gene expression system in *C. merolae* by inserting *lacO* at an appropriate position within a promoter. However, since there was no detailed structural or functional information available for the promoters of any genes in *C. merolae*, we attempted to apply the human interleukin-2 core (*cIL2*) promoter, for which detailed information, such as the position of the TATA box, is available (Weaver et al. 2007). Because *cIL2* promoter does not function on its own and requires specific transcription factors and regulatory elements to drive gene expression effectively (Weaver et al. 2007), we adopted a fusion of zinc finger homeodomain 1 (ZFHD1)-binding sequences (ZBS), consisting of 12 tandemly connected copies of the binding sequence, and *cIL2* promoter, which was modified from Amara et al. (1997) and the pZFHD1-2 vector (TAKARA). This system was originally designed for human cells, where the addition of rapamycin induces the formation of a heterodimer between ZFHD1-containing protein and another protein, thereby activating transcription (Amara et al. 1997). However, as shown below, we unexpectedly found that in *C. merolae*, ZFHD1 alone can activate gene transcription under the *ZBS*-*cIL2* promoter.

To test whether *ZBS*-*cIL2* promoter works in *C. merolae*, 2 strains, referred to as Z*BS-cIL2p* and *ZBS-cIL2p*  *+*  *ZFHD1 HA*, were generated (Supplementary Fig. S1A). In both strains, the *orf* encoding Venus protein, a fluorescent reporter, was located downstream of *ZBS-cIL2* promoter. In addition, in *ZBS-cIL2p*  *+*  *ZFHD1 HA* strain, ZFHD1, a chimeric transcription factor composed of the human Zif268 zinc finger domain and the human Oct-1 homeodomain (Pomerantz et al. 1995), was constitutively expressed to activate *ZBS-cIL2* promoter to transcribe *Venus*. For immunological detection, a 3×HA tag was fused to the C-terminus of ZFHD1 (ZFHD1 HA) (Supplementary Fig. S1A and Table S1). As a result, immunoblotting showed Venus was expressed in *ZBS-cIL2p*  *+*  *ZFHD1 HA* strain but not in *ZBS-cIL2p* strain (Supplementary Fig. S1B), demonstrating that the *ZBS-cIL2* promoter activated by ZFHD1 is able to express a gene in *C. merolae*.

**Figure 1.**
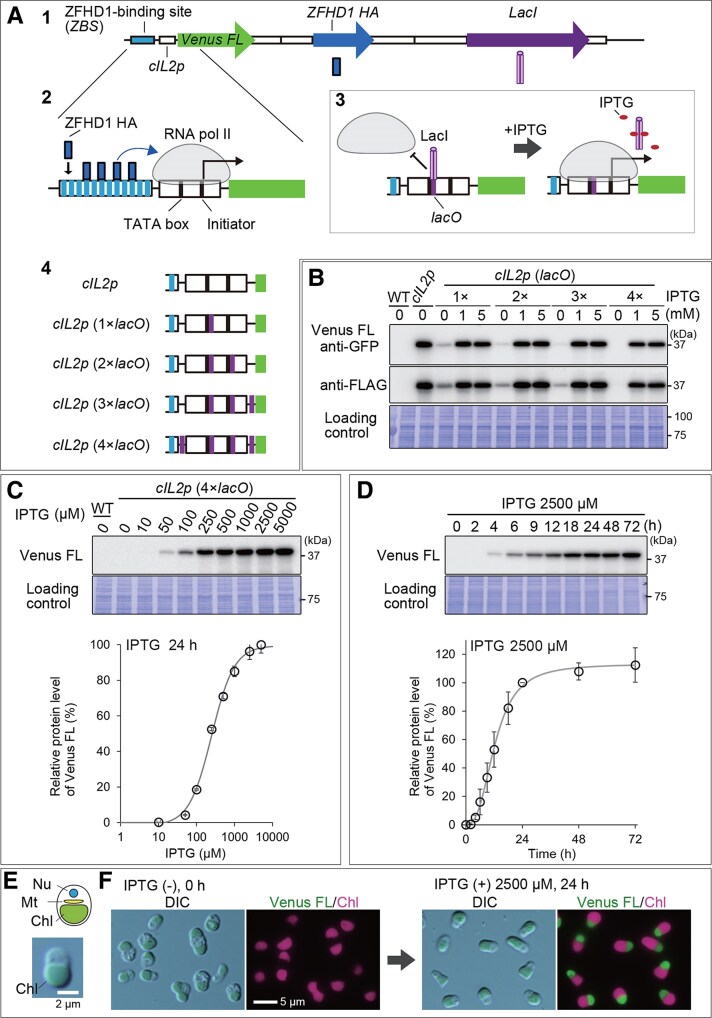
An IPTG-inducible gene expression system in *C. merolae* based on the ZFHD1-activated human *IL-2* core promoter transcriptional system. **A)** Schematic representation of the system. (1) Construction of the *ZBS-cIL2p-Venus FL* strain. Three gene expression cassettes were integrated into a chromosomal neutral site: (i) A gene encoding Venus tagged with 10×FLAG tags (*Venus FL*) under the control of a synthetic promoter composed of 12×ZFHD1-binding sequences (*ZBS*) and *IL-2* core promoter sequence (*cIL2p*); (ii) A gene encoding the transcription factor ZFHD1 tagged with 3×HA tags (*ZFHD1 HA*) constitutively expressed by *APCC* promoter; and (iii) a gene encoding LacI repressor constitutively expressed by *eEF1A* promoter. (2) ZFHD1 HA specifically binds to its target *ZBS* sequence and drives the transcription of *Venus FL* via c*IL2p*. (3) LacI repressor tightly binds to the *lacO* operator sequences inserted into or positioned adjacent to *cIL2p*, thereby competitively inhibiting RNA polymerase (RNA pol II) access to c*IL2p*. In the presence of IPTG, LacI dissociates from the *lacO* sequences, allowing RNA pol II to initiate transcription of *Venus FL*. (4) The structure of *cIL2p* and the positions of the *lacO* insertions examined in this study. Up to 4 *lacO* sequences (purple) were inserted into or positioned adjacent to *cIL2p*: the first *lacO* was placed immediately after the TATA box, the second was inserted just after the initiator sequence, the third was positioned 22 nucleotides upstream of the start codon of *Venus FL*, and the fourth was located immediately upstream of the *cIL2p* sequence. The detailed sequences are shown in Supplementary Fig. S2 and Supplementary Table S1. The construct was integrated into the upstream region of the chromosomal *URA* locus, which served as a neutral site, by homologous recombination. **B)** Immunoblotting with anti-GFP and anti-FLAG antibodies showing the *lacO* number-dependent suppression and IPTG-dependent induction of Venus FL expression (37 kDa) as a reporter. Cultures of *ZBS-cIL2p* or those with 1×, 2×, 3×, or 4× *lacO* insertions were supplemented with none, 1 mm, or 5 mm IPTG for 24 h. The wild-type (WT) culture served as a control. The Coomassie Brilliant Blue (CBB)-stained PVDF membrane is shown as a loading control. **C)** Immunoblotting showing the effect of IPTG at different concentrations on Venus FL expression. Cultures of *ZBS-cIL2p* (4×*lacO*) were treated with IPTG at concentrations ranging from 0 to 5,000 µM for 24 h. Immunoblotting with the anti-GFP antibody shows a dose-dependent increase in Venus FL protein levels. WT served as a control. The CBB-stained PVDF membrane is shown as a loading control. The accompanying graph shows relative Venus FL protein levels, calculated based on the band density in the immunoblot (the level at 5,000 µM IPTG was defined as 100%). The plots and error bars represent the averages and Sds of 3 biological replicates. **D)** Kinetics of the Venus FL protein level after IPTG induction in the *ZBS-cIL2p* (4×*lacO*) strain. The culture was treated with 2,500 µM IPTG, and the Venus FL protein level was monitored for 72 h by immunoblotting with the anti-GFP antibody. The CBB-stained PVDF membrane is shown as a loading control. Venus FL protein levels are presented in the accompanying graph, as in **(C)**. **E)** Schematic illustration of a *C. merolae* cell, along with differential interference contrast (DIC) and fluorescent images of *ZBS-cIL2p* (4×*lacO*) cells. The schematic illustration includes the nucleus (Nu), a single mitochondrion (Mt), and a single chloroplast (Chl) within the cell. A typical DIC image of a G1-phase cell is shown below the schematic, with a scale bar representing 2 µm. **F)** DIC and fluorescent images of *ZBS-cIL2p* (4×*lacO*) cells before and 24 h after the addition of 2,500 µM IPTG. Green and magenta represent the fluorescence of Venus FL and chloroplast (chlorophyll), respectively. The scale bar represents 5 µm.

Then, to test whether the *lacO*–LacI interaction reversibly inhibits the function of the *cIL2* promoter in *C. merolae*, we first generated 2 strains, designated as ZBS-*cIL2p*-*Venus FL* and ZBS-*cIL2p* (1*×lacO*)-*Venus FL* (Fig. 1A). In both strains, a gene cassette expressing Venus tagged with a 10×FLAG tag at its C-terminus (Venus FL), depending on *ZBS-cIL2* promoter and along with constructs constitutively expressing ZFHD1 tagged with a 3×HA tag under the *APCC* promoter and LacI under the *eEF1A* promoter, was integrated into a chromosomal neutral site. Additionally, in the *ZBS-cIL2p* (1*×lacO*)*-Venus FL* strain, a single *lacO* sequence was inserted immediately downstream of the TATA box of *cIL2* promoter sequence (Supplementary Fig. S2). The 10×FLAG tag attached to Venus was intended to enable highly sensitive detection of leaky protein expression.

In the absence of IPTG, immunoblotting showed that the Venus FL level is substantially reduced in the ZBS-*cIL2p* (1*×lacO*)-*Venus FL* strain compared to ZBS-*cIL2p-Venus FL* strain, indicating that the insertion of *lacO* inhibited *ZBS-cIL2* promoter activity (Fig. 1B). When 1 or 5 mm IPTG was added to the cultures, Venus FL expression in the *ZBS-cIL2p* (1*×lacO*)-*Venus FL* strain recovered within 24 h to a level comparable to that in the *ZBS-cIL2p-Venus FL* strain (Fig. 1B). Although there was still a slight amount of leaky expression of the target gene (in this case, Venus FL) in the absence of IPTG, these results indicated that the basic design of the IPTG-inducible system in *C. merolae* was appropriate. Thus, to reduce leaky expression, we increased the number of *lacO* insertions to 4, as previously tested in mouse and human cells (Hu and Davidson 1987; Myung et al. 2020).

Additional second, third, and fourth *lacO* sequences were inserted immediately downstream of the transcription initiator, as well as in the downstream and upstream flanking regions of the *cIL2* promoter, respectively (Fig. 1A, Supplementary Fig. S2). These strains were designated *ZBS-cIL2p* (2*×lacO*)*-Venus FL, ZBS-cIL2p* (3*×lacO*)*-Venus FL,* and *ZBS-cIL2p* (4*×lacO*)*-Venus FL*, respectively. In the absence of IPTG, Venus FL protein levels were reduced in a manner dependent on the number of *lacO* insertions and were undetectable in the *ZBS-cIL2p* (4*×lacO*)*-Venus FL* strain by immunoblotting (Fig. 1B). When 1 or 5 mm IPTG was added to the cultures, Venus FL expression in these 3 strains with *lacO* insertions recovered within 24 h to a level comparable to that in the *ZBS-cIL2p-Venus FL* strain without *lacO* insertion (Fig. 1B). Thus, an IPTG-inducible gene expression system was successfully developed utilizing the heterologous ZFHD1 transcription factor, its binding sites (*ZBS*), and the *cIL2* promoter with 4 *lacO* insertions in *C. merolae* (Fig. 1, A and B).

Then, we determined an appropriate range of working concentrations for IPTG (Fig. 1C). The *ZBS-cIL2p* (4*×lacO*)-*Venus FL* culture was treated with various IPTG concentrations ranging from 0 to 5,000 µM for 24 h. Immunoblotting showed that the Venus FL protein level increased in a dose-dependent manner from 50 to 2,500 µM, reaching saturation at 2,500 µM IPTG (Fig. 1C). Thus, the system has a wide dynamic range, allowing the protein level to be adjusted by changing the IPTG concentration.

Regarding the kinetics of IPTG-induced expression, with 2,500 µM IPTG, Venus FL protein was first detected 4 h after IPTG addition and reached a near-saturation level by 24 h (Fig. 1, D to F). This saturation level was maintained for at least 72 h after IPTG addition, suggesting that IPTG and its effect on *lacO*-LacI remain stable even in the acidic medium used to cultivate *C. merolae* (Fig. 1D).

### Design and establishment of an IPTG-inducible protein knockdown system in *C. merolae* utilizing a GFP nanobody-fused SKP1

We have recently developed a rapamycin-inducible protein knockdown system in *C. merolae* (Fujiwara et al. 2024). Rapamycin is a specific inhibitor of the mechanistic/mammalian target of rapamycin complex 1 (mTORC1) and inhibits its kinase activity by inducing heterodimerization between FK506-binding protein 12 (FKBP) and the FKBP12-rapamycin binding (FRB) domain of mTOR (Chen et al. 1995).

In the protein knockdown system, FKBP is fused to S-phase kinase-associated protein 1 (SKP1) or Cullin 1 (CUL1), which are components of the SKP1–CUL1–F-box (SCF) E3 ligase. A protein of interest is fused with the FRB domain. In the cell, upon rapamycin treatment, the protein of interest binds to FKBP-SKP1 or FKBP-CUL1, becomes ubiquitinated by the SCF E3 ligase, and is substantially degraded within 2 h (Fujiwara et al. 2024). This system enables quick and efficient targeted protein degradation. However, some limitations still remain, including (1) the short duration of the degradation effect, likely due to rapamycin degradation in the acidic medium required for *C. merolae* cultivation, and (2) limited but potentially significant side effects on cell physiology due to mTORC1 inhibition, depending on the application.

**Figure 2.**
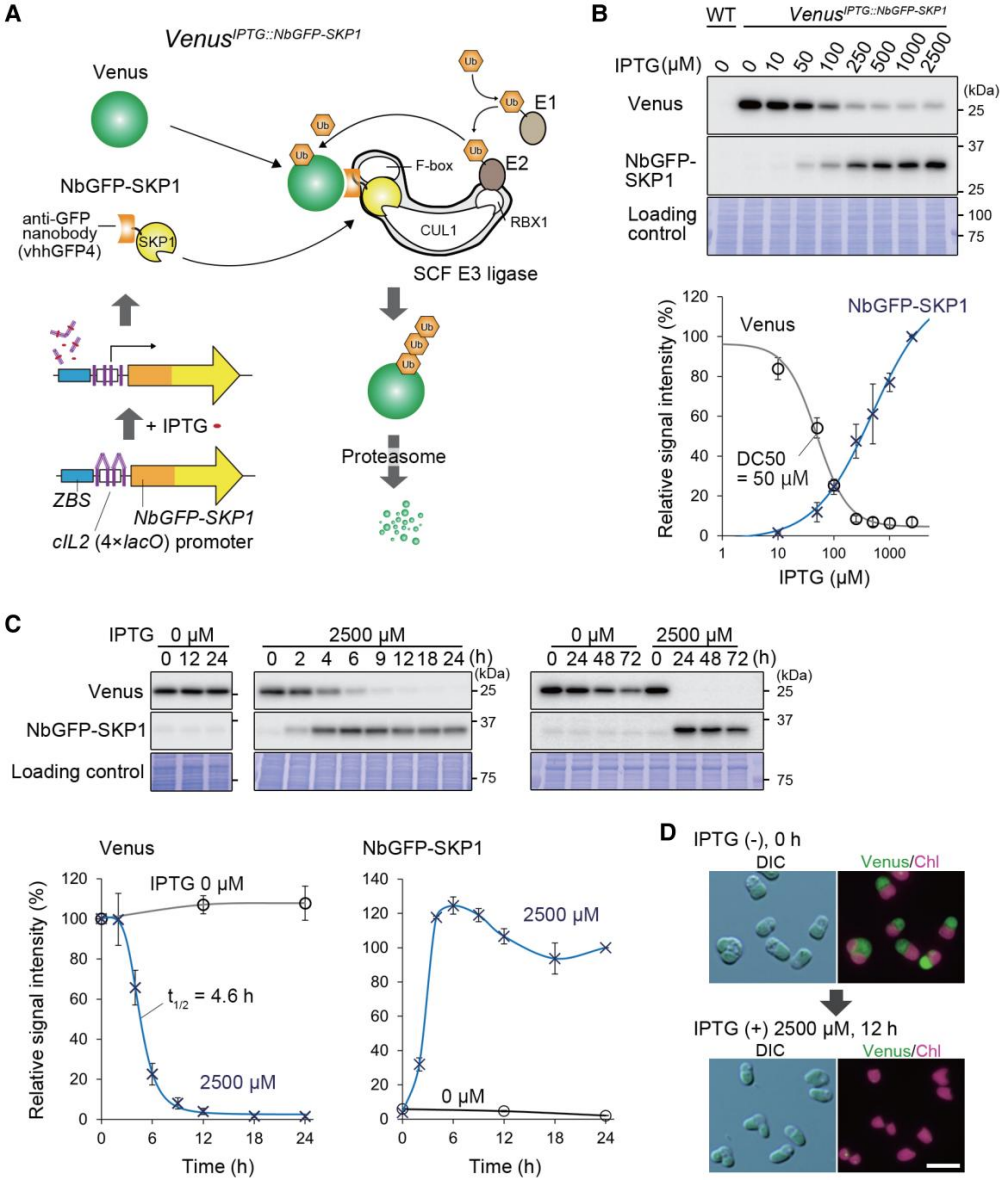
An IPTG-inducible protein knockdown system in *C. merolae* mediated by an anti-GFP nanobody conjugated with SKP1. **A)** Schematic representation of the system. Venus protein (a GFP variant) serves as the degradation target. The anti-GFP nanobody (vhhGFP4), conjugated with SKP1, a component of the SCF E3 ligase (NbGFP-SKP1), is designed as a targeted ubiquitination inducer that is expressed upon IPTG treatment. Upon IPTG treatment, NbGFP-SKP1 is expressed and binds to Venus, triggering its ubiquitination by the SCF E3 ligase and directing Venus for proteasomal degradation. To test the system, the *Venus^IPTG::NbGFP-SKP1^* strain was generated. This strain constitutively expresses Venus under the control of the *EFTU* promoter and inducibly expresses NbGFP-SKP1 under the *ZBS-cIL2p* (4×*lacO*) sequence upon IPTG treatment, from the upstream region of the *URA* locus (a chromosomal neutral site). The detailed sequences are shown in Supplementary Fig. S2 and Supplementary Table S1. **B)** Immunoblotting showing the effect of IPTG at different concentrations on NbGFP-SKP1 (32 kDa; detected with the anti-SKP1 antibody) expression and Venus (27 kDa; detected with the anti-GFP antibody) degradation. *Venus^IPTG::NbGFP-SKP1^* cultures were treated with IPTG at concentrations ranging from 0 to 5,000 μM for 24 h. The wild-type (WT) served as a control. The Coomassie Brilliant Blue (CBB) -stained PVDF membrane is shown as a loading control. The accompanying graph shows relative Venus and NbGFP-SKP1 protein levels, calculated based on the band density in the immunoblot (the Venus level at 0 µM IPTG and NbGFP-SKP1 at 2,500 μM IPTG were each defined as 100%.). The plots and error bars represent the averages and Sds of 3 biological replicates. The half-maximal degradation concentration (DC50) was calculated using Rodbard curve fitting in ImageJ (Schneider et al. 2012). **C)** Degradation kinetics of Venus after IPTG induction in the *Venus^IPTG::NbGFP-SKP1^* strain. The culture was treated with 2,500 µM IPTG, and Venus and NbGFP-SKP1 protein levels were monitored for 72 h by immunoblotting. The CBB-stained PVDF membranes are shown as a loading controls. Venus and NbGFP-SKP1protein levels are presented in the accompanying graphs, as in **(B)**, except that the Venus level at 0 h with each concentration of IPTG was defined as 100%, while the NbGFP-SKP1 level at 24 h with 2,500 µM IPTG was defined as 100%. The half-life (t_1/2_) was calculated using Rodbard curve fitting in ImageJ, while other fitting lines were generated in Excel. **D)** Differential interference contrast (DIC) and fluorescent images of the *Venus^IPTG::NbGFP-SKP1^*cells before and 12 h after the addition of 2,500 µM IPTG. Green and magenta represent the fluorescence of Venus and chloroplast (chlorophyll), respectively. The scale bar represents 5 µm.

To address these issues, we aimed to develop an IPTG-driven inducible protein knockdown system in *C. merolae* (Fig. 2). We generated a strain named *Venus^IPTG::NbGFP-SKP1^*, which constitutively expresses the Venus protein (a GFP variant) as a degradation target under the control of the elongation factor thermos-unstable (*EFTU*) promoter and inducibly expresses a GFP nanobody (vhhGFP4; Caussinus et al. 2012) fused with SKP1 (NbGFP-SKP1) under the *ZBS-cIL2* (4*×lacO*) promoter upon IPTG treatment (Fig. 2A). The inducible *NbGFP–SKP1* expression cassette was integrated into a chromosomal neutral site, distinct from the endogenous *SKP1* locus, ensuring that endogenous SKP1 expression remains unaffected. This system is an adaptation of the deGradFP system, which uses an F-box protein as the ubiquitination inducer (Caussinus et al. 2012). However, following our previous study (Fujiwara et al. 2024), we chose to use SKP1 instead. In this system in *C. merolae*, IPTG treatment induces NbGFP-SKP1 expression and its binding to Venus. The Venus protein is then ubiquitinated by the SCF E3 ligase and degraded by the proteasome (Fig. 2A).

To evaluate the system, the *Venus^IPTG::NbGFP-SKP1^* culture was treated with various concentrations of IPTG ranging 0 to 2,500 μM for 24 h. Immunoblotting showed a dose-dependent increase in the NbGFP-SKP1 protein level (Fig. 2B) and, inversely correlated with this, a dose-dependent reduction in the Venus protein level, reaching a minimum at 250 µM IPTG, with a half-maximal degradation concentration (DC₅₀) of 50 µM (Fig. 2B).

Then, we compared the Venus degradation efficiency between IPTG-induced NbGFP-CUL1 and NbGFP-SKP1, as FKBP-CUL1 exhibited higher degradation efficiency than FKBP-SKP1 in the previously developed rapamycin-inducible protein knockdown system (Fujiwara et al. 2024). For this comparison, we generated the *Venus^IPTG::NbGFP-CUL1^* strain, in which the GFP nanobody is fused to CUL1 instead of SKP1. When the *Venus^IPTG::NbGFP-SKP1^* and *Venus^IPTG::NbGFP-CUL1^* cultures were treated with 2,500 µM IPTG for 24 h, both exhibited similar degradation efficiency of the Venus protein (Supplementary Fig. S3). However, the *SKP1 orf* (510 bp) is much smaller than the *CUL1 orf* (3,042 bp), making it more convenient for constructing a protein knockdown system. Consequently, we proceeded with subsequent experiments using SKP1 in this study.

Next, we evaluated the kinetics of NbGFP-SKP1 and Venus in the *Venus^IPTG::NbGFP-SKP1^* culture following the addition of 2,500 µM IPTG (Fig. 2C), the concentration at which NbGFP-SKP1 reached its highest levels (Fig. 2B). In the absence of IPTG, NbGFP-SKP1 was nearly undetectable by immunoblotting, and Venus protein was constitutively expressed for 24 h (Fig. 2C). It should be noted that endogenous SKP1 is expressed constitutively, as shown later, and that the absence observed here refers only to the inducible NbGFP–SKP1. After IPTG addition, NbGFP-SKP1 was first detected at 2 h and reached its maximum level 6 h after the addition (Fig. 2C). Correspondingly, Venus protein levels began to decrease at 2 h and continued declining until reaching a minimum 12 h after IPTG addition, with a half-life (*t*_1/2_) of 4.6 h. This Venus degradation effect lasted for at least 72 h (Fig. 2C). Even in the absence of IPTG, a slight decrease in Venus levels was observed from 24 to 72 h after the start of the culture (Fig. 2C). This is possibly due to a decrease in the activity of the *EFTU* promoter caused by environmental changes, such as an increase in cell concentration and a decrease in the availability of nutrients, dissolved CO_2_, light, and cellular growth.

We also confirmed IPTG-induced degradation of the Venus protein by fluorescence microscopy (Fig. 2D). Thus, we successfully developed an IPTG-inducible protein knockdown system using NbGFP-SKP1 in *C. merolae*. To knock down an endogenous protein of interest, the target protein needs to be fused with GFP or a variant such as Venus via a knock-in approach.

### Another IPTG-inducible protein knockdown system in *C. merolae* utilizing the ALFA-tag and its nanobody

NbGFP can bind to a wide variety of GFP variants (∼27 kDa) with high affinity, exhibiting a dissociation constant (KD) of ∼1 nm (Kubala et al. 2010), which is the reason for its use in the inducible protein knockdown system. However, GFP variants are large compared to widely used epitope tags, and such a large tag may sometimes hinder the function of the fusion protein. Regarding nanobodies, the ALFA tag (15 amino acids, 1.9 kDa) and the anti-ALFA-tag nanobody (NbALFA) have been developed, and their interaction is exceptionally strong (KD = 26 pM) (Götzke et al. 2019); however, they have not yet been utilized in a protein knockdown system.

**Figure 3.**
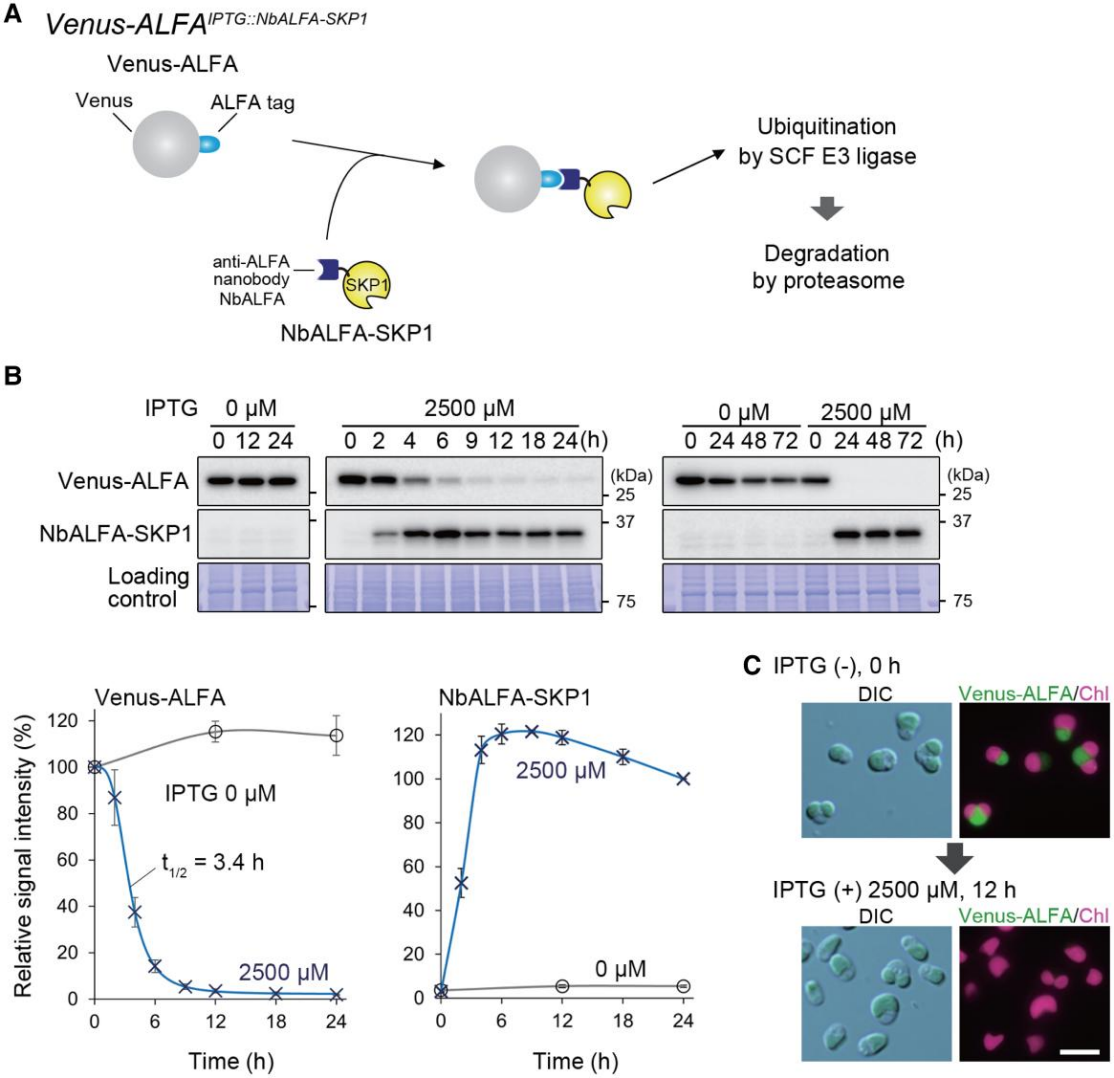
An IPTG-inducible protein knockdown system in *C. merolae* mediated by an anti-ALFA tag nanobody conjugated with SKP1. **A)** Schematic representation of the system. Venus protein tagged with ALFA tag (15 amino acids, 1.9 kDa) at the C-terminus (Venus-ALFA) serves as a degradation target. An anti-ALFA nanobody, conjugated with SKP1, a component of the SCF E3 ligase (NbALFA-SKP1), is designed as a targeted ubiquitination inducer that is expressed upon IPTG treatment. Upon IPTG treatment, NbALFA-SKP1 is expressed and binds to Venus-ALFA, triggering its ubiquitination by the SCF E3 ligase and directing Venus-ALFA for proteasomal degradation. To test the system, the *ALFA^IPTG::NALFA-SKP1^* strain was generated. This strain constitutively expresses Venus-ALFA under the control of the EFTU promoter and inducibly expresses NbALFA-SKP1 under the *ZBS-cIL2p* (4×*lacO*) sequence upon IPTG treatment from the upstream region of the *URA* locus (a chromosomal neutral site). The detailed sequences are shown in Supplementary Fig. S2 and Supplementary Table S1. **B)** Degradation kinetics of Venus-ALFA after IPTG induction in the *ALFA^IPTG::NALFA-SKP1^* culture. The culture was treated with 2,500 µM IPTG, and Venus-ALFA (29 kDa; detected with the anti-GFP antibody) and NbALFA-SKP1 (33 kDa; detected with the anti-SKP1 antibody) protein levels were monitored for 72 h by immunoblotting. The Coomassie Brilliant Blue (CBB) -stained PVDF membranes are shown as loading controls. The accompanying graphs show relative Venus-ALFA and NbGFP-SKP1 protein levels, calculated based on the band density in the immunoblot (the Venus-ALFA level at 0 h under each IPTG concentration condition was defined as 100%, while the NbALFA-SKP1 level at 24 h with 2,500 µM IPTG was defined as 100%). The plots and error bars represent the averages and Sds of 3 biological replicates. The *t*_1/2_ was calculated using Rodbard curve fitting in ImageJ, while other fitting lines were generated in Excel. **C)** Differential interference contrast (DIC) and fluorescent images of the *ALFA^IPTG::NALFA-SKP1^* cells before and 12 h after the addition of 2,500 µM IPTG. Green and magenta represent the fluorescence of Venus-ALFA and chloroplast (chlorophyll), respectively. The scale bar represents 5 µm.

To develop a protein knockdown system using the ALFA tag and NbALFA-SKP1 instead of Venus and NbGFP, we generated a strain named *Venus-ALFA^IPTG::NbALFA-SKP1^*. This strain constitutively expresses Venus-ALFA under the *EFTU* promoter and inducibly expresses NbALFA-SKP1 under the *ZBS-cIL2* (4*×lacO*) promoter upon IPTG addition (Fig. 3A). When 2,500 µM IPTG was added to the *Venus-ALFA^IPTG::NbALFA-SKP1^* culture, NbALFA-SKP1 was first detected at 2 h and almost reached its maximum level 4 h after the addition. Correspondingly, the Venus-ALFA protein level began to decrease soon after IPTG addition and reached its minimum at 9 h, with a half-life (*t*_1/2_) of 3.4 h. The effect of IPTG on Venus-ALFA degradation lasted for at least 72 h (Fig. 3B). We also confirmed the degradation of Venus-ALFA upon IPTG addition by fluorescence microscopy (Fig. 3C). Thus, we successfully developed an IPTG-inducible protein knockdown system utilizing the small ALFA tag and NbALFA-SKP1.

### Design and establishment of an estradiol-inducible gene expression system in *C. merolae*

**Figure 4.**
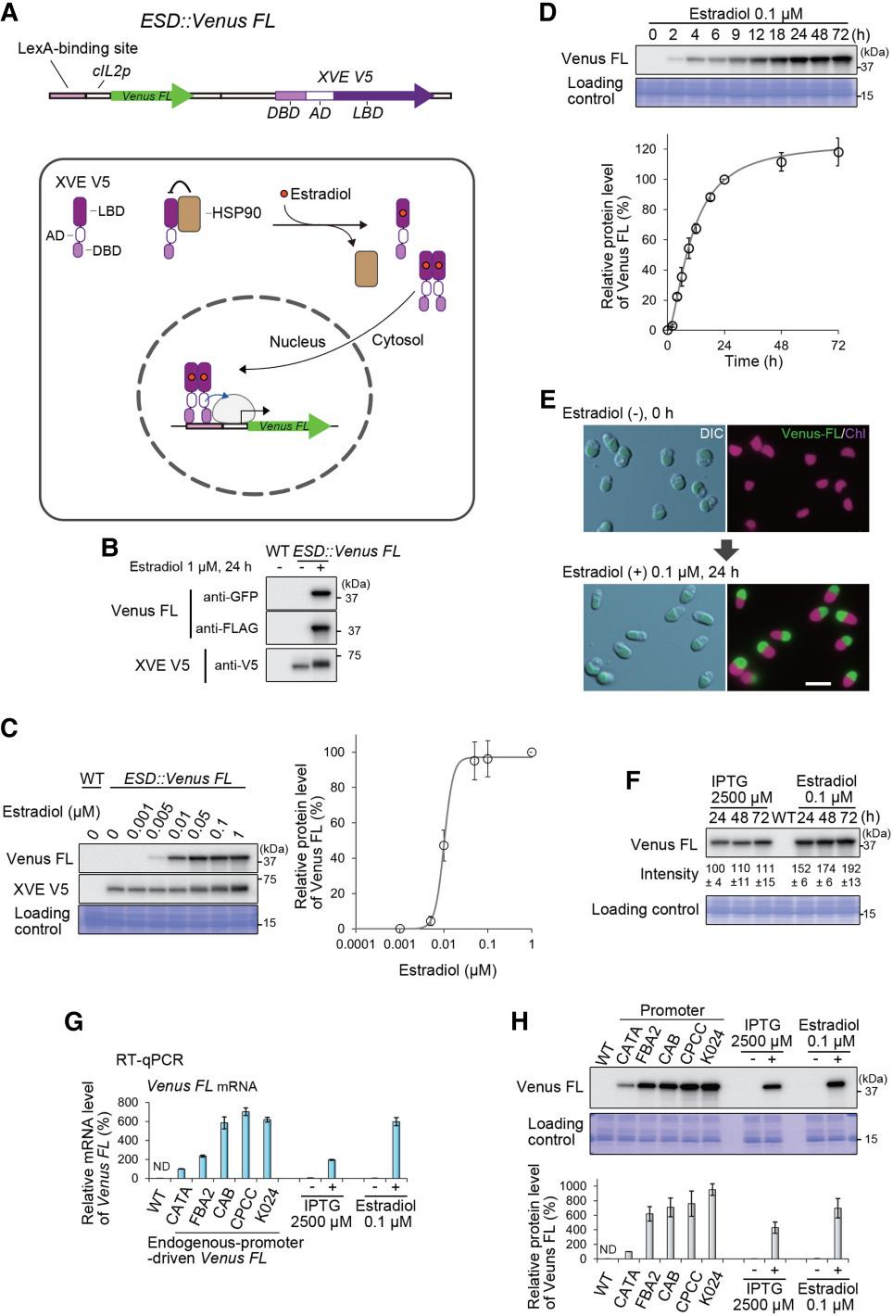
An estradiol-inducible gene expression system in *C. merolae* based on the chimeric transcription activator XVE and the LexA-binding sequence. **A)** Schematic representation of the system. To test the system, 2 gene expression cassettes were integrated into the upstream region of the *URA* locus (a chromosomal neutral site), generating the *ESD::Venus FL* strain: (i) a gene encoding Venus tagged with 10×FLAG tags (*Venus FL*) under the control of a synthetic promoter composed of 4×LexA operator sequences (LexA-binding site) and the *IL-2* core promoter sequence (*cIL2p*); and (ii) a gene encoding the artificial chimeric transcription factor XVE tagged with 3×V5 tags (*XVE V5*), constitutively driven by the *CMK024C* promoter. The detailed sequences are shown in Supplementary Fig. S2 and Supplementary Table S1. XVE is composed of 3 domains: the LexA DNA-binding domain from *Escherichia coli* (DBD), the VP16 transcriptional activation domain from the herpes simplex virus (AD), and the ligand-binding domain from the human estrogen receptor (LBD). In the absence of estradiol, XVE V5 binds to the HSP90 complex via the LBD, sequestering it in the cytoplasm and maintaining its inactive state. In the presence of estradiol, its binding to the LBD domain of XVE V5 releases XVE V5 from HSP90, promotes LBD homo-dimerization, and facilitates its translocation to the nucleus, where it activates transcription through the binding of the DBD domain to the LexA-binding site. **B)** Immunoblotting with anti-GFP and anti-FLAG antibodies showing the expression of Venus FL (37 kDa) in the *ESD::Venus FL* strain 24 h after the addition of 1 µM estradiol to the culture. Constitutive expression of XVE V5 (58 kDa) in the strain, regardless of the presence or absence of estradiol, was also confirmed by immunoblotting with the anti-V5 antibody. The wild-type (WT) culture served as a control. **C)** Immunoblotting showing the effect of estradiol at different concentrations on Venus FL expression in the *ESD::Venus FL* strain. The cultures were treated with estradiol at concentrations ranging from 0 to 1 µM for 24 h. Immunoblotting with the anti-GFP antibody shows a dose-dependent increase in the Venus FL protein level. Constitutive expression of XVE V5 was confirmed using anti-V5 antibodies. WT served as a control. The Coomassie Brilliant Blue (CBB) -stained PVDF membrane is shown as a loading control. The accompanying graph shows relative Venus FL protein levels, calculated based on the band density in the immunoblot (the level at 1 µM estradiol was defined as 100%). The plots and error bars represent the averages and Sds of 3 biological replicates. The curve was generated using Rodbard curve fitting in ImageJ. **D)** Kinetics of the Venus FL protein level after estradiol induction in the *ESD::Venus FL* strain. The culture was treated with 0.1 µM estradiol, and the Venus FL protein level was monitored for 72 h by immunoblotting with the anti-GFP antibody. The CBB-stained PVDF membrane is shown as a loading control. Venus FL protein levels are presented in the accompanying graph, as in **(C)**. **E)** Differential interference contrast (DIC) and fluorescent images of *ESD::Venus FL* cells before and 24 h after the addition of 0.1 µM estradiol. Green and magenta represent the fluorescence of Venus FL and chloroplast (chlorophyll), respectively. The scale bar represents 5 µm. **F)** Comparison of the maximum Venus FL protein levels between the IPTG- and estradiol-inducible expression systems. The *ZBS-cIL2p* (4×*lacO*) and *ESD::Venus FL* cultures were treated with 2,500 µM IPTG and 0.1 µM estradiol, respectively, for 24, 48, and 72 h. WT served as a control. The values represent relative Venus FL protein levels, calculated based on the band density in the immunoblot. The averages and Sds of 3 biological replicates are indicated. The value at 24 h in *ZBS-cIL2p* (4×*lacO*) was defined as 100%. The CBB-stained PVDF membrane is shown as a loading control. **G)** RT–qPCR analysis of *Venus FL* mRNA levels in IPTG- and estradiol-inducible systems and in strains with *Venus FL* driven by endogenous promoters (*CATA*, *FBA2, CAB, CPCC,* and *K024*). The wild-type strain (WT) served as a negative control. IPTG with its vehicle (distilled water) and estradiol with its vehicle (acetone) were added to the corresponding strains on day 0, and all cultures were harvested on day 3 for total RNA extraction. RT–qPCR was performed using primers targeting the *Venus FL* ORF, with *DRP3* ORF as an internal control. *Venus FL* values were normalized to those driven by the CATA promoter (set to 100%). Technical triplicates were performed, and data are presented as the mean ± Sd. Values for the WT are indicated as ND (not detected). **H)** Immunoblot analysis of Venus FL protein levels using an anti-GFP antibody in the same set of samples as in **(G)**. Cultures were harvested on day 3 for protein extraction. Protein levels were normalized to those driven by the *CATA* promoter (set to 100%). Technical triplicates of immunoblot analyses were performed, and data are presented as the mean ± Sd. The signal for the WT was not detected (ND). The CBB-stained membrane is shown as a loading control.

Estradiol-inducible gene expression systems have been developed in flowering plants and mosses (Zuo et al. 2000; Kubo et al. 2013). These systems basically consist of a chimeric transcription factor, XVE, its specific binding DNA sequence (LexA-binding site; *LBS*), and the downstream Cauliflower mosaic virus (CaMV) 35S core promoter. The artificial XVE protein is composed of 3 functional domains: the DNA-binding domain of the LexA protein from *Escherichia coli* (DBD), the transcriptional activation domain of the VP16 protein from a herpes simplex virus (AD), and the ligand (estradiol)-binding domain of the estrogen receptor from a mammal (LBD) (Fig. 4A). In the absence of estradiol, XVE is sequestered in the cytosol by the endogenous chaperone complex, including the HSP90 protein (Noddings et al. 2022). Upon estradiol binding to the ligand-binding domain, XVE dissociates from the HSP90 complex, forms a homodimer, translocates into the nucleus, and acts as an activated transcription factor by binding to the LBS and activating downstream gene expression from the CaMV 35S core promoter (Zuo et al. 2000) (Fig. 4A).

In this study, we planned to introduce the XVE system into *C. merolae* and evaluate its functionality. However, the CaMV 35S promoter has been reported to be nonfunctional in *C. merolae* (Watanabe et al. 2011). Thus, we utilized *cIL2* promoter, which, as shown above, has been demonstrated to be functional in *C. merolae*, instead of the CaMV 35S core promoter (Fig. 4A). Based on this concept, we generated a strain referred to as *ESD::Venus FL*. This strain constitutively expresses XVE V5 under the control of the *CMK024C* promoter. For immunodetection, a 3×V5 tag was fused to the C-terminus of XVE. Additionally, the strain contains a gene cassette consisting of *LBS* (4 copies of the LexA operator sequences; Supplementary Fig. S4) upstream of *cIL2* promoter, which is designed to drive Venus FL expression as a reporter upon estradiol treatment (Fig. 4A).

As expected, Venus FL protein was undetectable in the absence of estradiol in the *ESD::Venus FL* culture. In contrast, in the presence of 1 µM estradiol, Venus FL expression was detected in the strain by immunoblotting. As designed, XVE V5 was expressed in the strain regardless of estradiol presence, but a band shift was observed upon estradiol treatment (Fig. 4B). This shift is possibly caused by phosphorylation, as the LBD of the estrogen receptor is regulated by various signaling pathways through phosphorylation (Anbalagan and Rowan 2015). In estrogen receptor α (ERα), phosphorylation of Tyr537 within the LBD stabilizes receptor dimerization and thereby facilitates transcriptional activation (Arnold et al. 1995). XVE likewise retains the corresponding Tyr425 in the LBD, and if this residue is phosphorylated in *C. merolae*, it may contribute to the stability of dimer formation. In parallel, the LexA DBD of XVE itself can dimerize (Zuo et al. 2000) and binds to the LexA operator, which is organized as an inverted repeat sequence. Thus, in addition to dimerization mediated by the LexA DBD, phosphorylation of the ERα-derived LBD could further stabilize the overall dimer. Such stabilization is expected to improve the specificity of DNA binding, which in turn could enhance the efficiency of transcriptional activation at the LexA operator.

To determine the working concentration of estradiol, the *ESD::Venus FL* culture was treated with varying estradiol concentrations ranging from 0 to 1 µM for 24 h. Immunoblotting (with the anti-GFP antibody) showed that the Venus FL protein was expressed at detectable levels with 0.005 µM or higher estradiol, and the expression level reached near saturation at approximately 0.02 µM estradiol (Fig. 4C). Thus, the protein level can be regulated by adjusting the estradiol concentration in this *C. merolae* system. However, the dynamic range of estradiol-induced expression is relatively narrow compared to IPTG-inducible expression, which limits its flexibility for precise regulation. A prominent band shift of XVE V5 was observed at estradiol concentrations of 0.05 µM and above.

Regarding the kinetics, upon the addition of 0.1 µM estradiol, a concentration that achieved fully saturated induction (Fig. 4C), the Venus protein level became detectable at 2 h and reached near saturation at 24 h. The effect of estradiol persisted for at least 72 h (Fig. 4D). We also confirmed the induction of Venus FL expression by estradiol treatment using fluorescence microscopy (Fig. 4E). Thus, we successfully developed an estradiol-inducible gene expression system in *C. merolae*.

So far, we have developed IPTG- and estradiol-inducible gene expression systems. Then, we compared the expression levels of Venus FL protein as the target protein in both systems. To this end, the *ZBS-cIL2p* (4*×lacO*)-*Venus FL* and *ESD::Venus FL* cultures were treated with 2,500 µM IPTG or 0.1 µM estradiol for 72 h. Immunoblotting showed that the estradiol system yielded 1.5- to 1.7-fold higher Venus FL protein levels from 24 to 72 h compared to IPTG system (Fig. 4F).

To further assess the induced gene expression levels, we quantified Venus FL mRNA and protein accumulation upon induction, relative to expression driven by endogenous promoters, including those of catalase (*CATA*), fructose-1,6-bisphosphate aldolase (*FBA2*), chlorophyll a/b-binding protein (*CAB*), phycocyanin-associated rod linker protein (*CPCC*), and a gene encoding a protein of unknown function (CMK024C; *K024*). These promoters are predicted to exhibit strong transcriptional activity based on previous transcriptome data (Fujiwara et al. 2020) and have been utilized to drive the expression of exogenous genes such as selection markers (Fujiwara et al. 2017, 2021). In the present study, their transcript abundances ranked 29th, 14th, 4th, second, and first, respectively, among all nuclear genes examined (Supplementary Table S2; WT with vehicle in the IPTG experiment). IPTG or estradiol was added at culture initiation in the respective inducible strains, and expression levels were compared on day 3 for the 2 inducible and 5 endogenous promoter strains. RT–qPCR analysis showed that the relative *Venus FL* mRNA levels driven by the endogenous promoters were largely consistent with the trends observed in the RNA-seq data (Fig. 4G; Supplementary Table S2). The IPTG-induced *Venus FL* mRNA level was comparable to that driven by the *FBA2* promoter, whereas the estradiol-induced *Venus FL* mRNA reached a level similar to those driven by the *CAB* and *K024* promoters (Fig. 4G).

To examine whether the mRNA expression trends were also reflected at the protein level, we analyzed Venus FL protein accumulation. Immunoblotting with an anti-GFP antibody showed that the relative Venus FL protein levels among the endogenous promoter strains were broadly consistent with the mRNA levels, although a discrepancy was observed between *CPCC* and *K024* (Fig. 4H). This discrepancy is likely attributable to differences in the 5′ untranslated regions (5′ UTRs) affecting translational efficiency, as well as to the high stability of Venus FL protein (Corish and Tyler-Smith 1999). The IPTG-induced Venus FL protein level was higher than that in the *CATA* strain but approximately two-thirds of that in the *FBA2* strain, whereas the estradiol-induced Venus FL protein reached a level similar to those in the *FBA2, CAB*, and *CPCC* strains (Fig. 4H). These results demonstrate that both inducible systems can achieve expression levels approaching those driven by strong endogenous promoters, thereby providing a useful method for overexpression analyses of genes of interest.

### Design and establishment of an estradiol-inducible protein knockdown system in *C. merolae* using the ALFA tag and its nanobody

**Figure 5.**
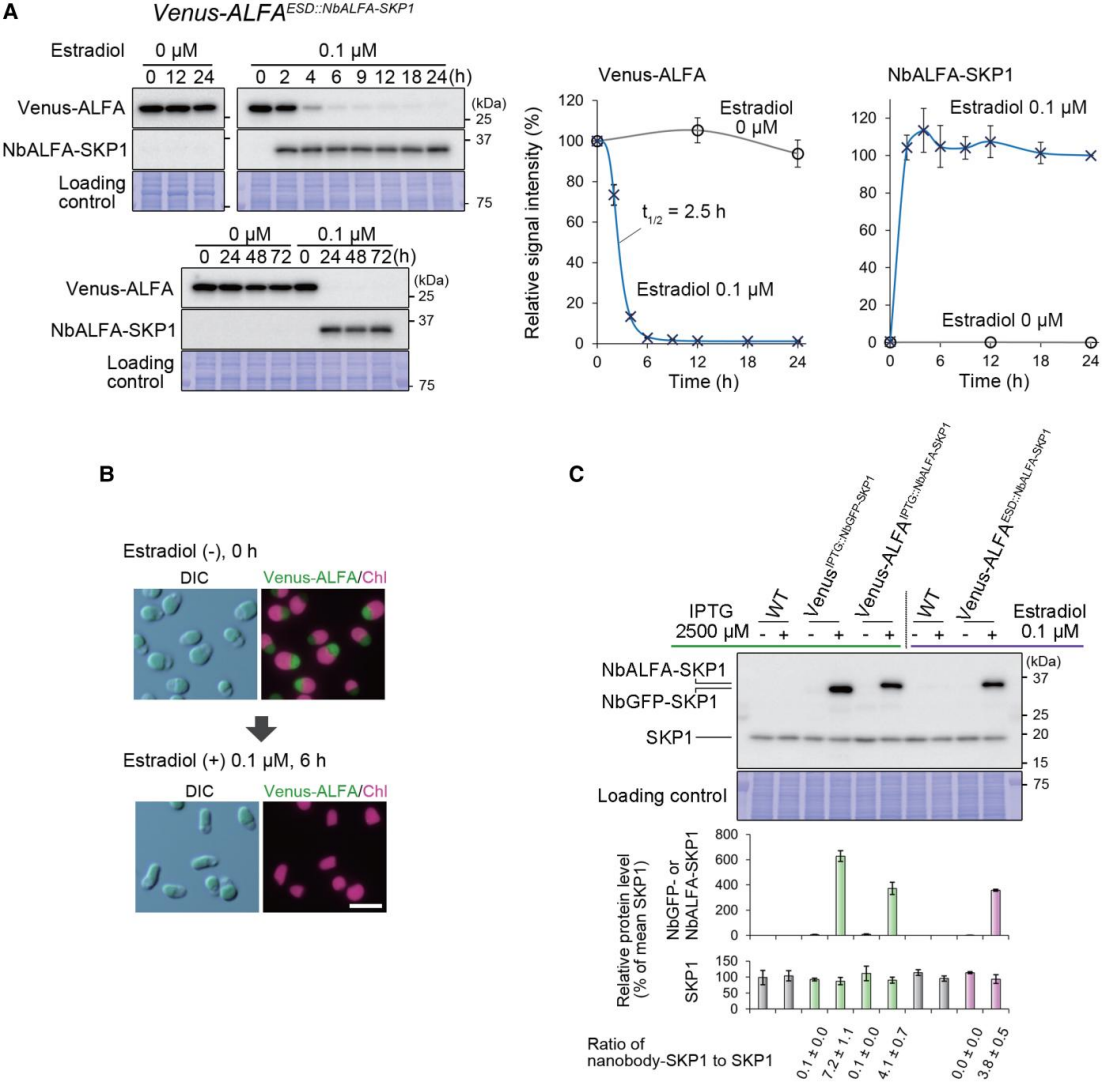
An estradiol-inducible protein knockdown system in *C. merolae* mediated by an anti-ALFA tag nanobody conjugated with SKP1. To test the system, the *Venus-ALFA^ESD::NbALFA-SKP1^* strain was generated. This strain constitutively expresses Venus-ALFA as a degradation target under the control of the *EFTU* promoter and inducibly expresses NbALFA-SKP1 under the LexA-binding site and *cIL2p* upon estradiol treatment, from the upstream region of the *URA* locus (a chromosomal neutral site). The detailed sequences are shown in Supplementary Fig. S2 and Supplementary Table S1. **A)** Kinetics of NbALFA-SKP1 accumulation and Venus-ALFA degradation after estradiol induction in the *Venus-ALFA^ESD::NbALFA-SKP1^* strain. The culture was treated with 0.1 µM estradiol, and Venus-ALFA (27 kDa) and NbALFA-SKP1 (33 kDa) protein levels were monitored for 72 h by immunoblotting with anti-GFP and anti-SKP1 antibodies, respectively. The Coomassie Brilliant Blue (CBB)-stained PVDF membranes are shown as loading controls. The accompanying graphs show relative Venus-ALFA and NbALFA-SKP1 protein levels, calculated based on band density in the immunoblot (the Venus-ALFA level at 0 h was defined as 100%, while the NbALFA-SKP1 level at 24 h with 0.1 μM estradiol was defined as 100%). The plots and error bars represent the averages and Sd of 3 biological replicates. The half-life (*t*_1/2_) was calculated using Rodbard curve fitting in ImageJ, while other fitting lines were generated in Excel. **B)** Differential interference contrast (DIC) and fluorescent images of the *Venus-ALFA^ESD::NbALFA-SKP1^* cells before and 6 h after the addition of 0.1 µM estradiol. Green and magenta represent the fluorescence of Venus-ALFA and chloroplast (chlorophyll), respectively. The scale bar represents 5 µm. **C)** Ratio of induced NbGFP- and NbALFA-SKP1 to endogenous SKP1. Immunoblotting of wild type, *Venus^IPTG::NbGFP-SKP1^*, *Venus^IPTG::NbALFA-SKP1^,* and *Venus^ESD::NbALFA-SKP1^* was performed using an anti-SKP1 antibody in the absence or presence of inducing reagents. The CBB-stained membrane served as a loading control. The predicted molecular masses were 19 kDa for endogenous SKP1 and 32 kDa for NbGFP-SKP1 and NbALFA-SKP1. The accompanying bar graph shows the quantified protein levels, with the mean endogenous SKP1 level across all samples set to 100%. Values represent the mean of 3 technical replicates of immunoblotting, and error bars indicate Sd.

To apply the estradiol-inducible gene expression system to targeted protein knockdown, we generated a strain referred to as *Venus-ALFA^ESD::NbALFA-SKP1^*, which constitutively expresses Venus under the control of the *EFTU* promoter and inducibly expresses NbALFA-SKP1 under the *LBS-cIL2* promoter upon estradiol treatment. To test the system, the *Venus-ALFA^ESD::NbALFA-SKP1^* culture was treated with 0.1 µM estradiol for 24 h. Immunoblotting showed that a decrease in Venus-ALFA levels became detectable 2 h after estradiol addition and reached near saturation at 6 h, with a half-life (*t*_1/2_) of 2.5 h. This degradation effect persisted for at least 72 h (Fig. 5A). We also confirmed the disappearance of Venus-ALFA fluorescence 6 h after the addition of estradiol using fluorescence microscopy (Fig. 5B).

Each inducible expression cassette of nanobody-fused *SKP1* was introduced into a neutral genomic site distinct from the endogenous *SKP1* locus. We then compared the protein levels of endogenous SKP1 and induced nanobody-fused SKP1 to evaluate whether the induced proteins were expressed at levels sufficient to substitute for endogenous SKP1 within the SCF complex. Upon induction with 2,500 µM IPTG or 0.1 µM estradiol for 24 h, NbGFP–SKP1 and NbALFA–SKP1 accumulated to 7.2-, 4.1-, and 3.8-fold the levels of endogenous SKP1 in *Venus^IPTG::NbGFP–SKP1^*, *Venus-ALFA^IPTG::NbALFA–SKP1^*, and *Venus-ALFA^ESD::NbALFA–SKP1^* strains, respectively (Fig. 5C). Assuming that the efficiency of incorporation of nanobody-fused SKP1 into the SCF complex is comparable to that of endogenous SKP1, the induced nanobody-fused SKP1 proteins appear to be produced at sufficiently high levels to effectively replace endogenous SKP1, thereby enabling efficient targeted protein knockdown.

### Effect of IPTG and estradiol removal on turning off induced gene expression in *C. merolae*

Next, we evaluated whether the expression of a target gene induced by IPTG or estradiol is effectively turned off when these chemicals were removed from the culture. To this end, the *ZBS-cIL2p* (4*×lacO*)*-Venus FL* and *ESD::Venus FL* cultures were treated with 2,500 µM IPTG and 0.1 µM estradiol, respectively, for 24 h. Then, IPTG and estradiol were removed from the respective cultures by centrifugation and resuspension of the cell pellets into fresh medium 3 times, and the cells were further cultured without IPTG or estradiol. Next, the change in *Venus FL* mRNA level was examined by quantitative reverse-transcription PCR (qRT-PCR) at 0, 1, 4, and 24 h after removal. In the *ZBS-cIL2p* (4*×lacO*)-*Venus FL* culture, the *Venus FL* mRNA level gradually decreased, reaching 40% of the induced state at 24 h, while in the *ESD::Venus FL* culture, they dropped to 2% by 4 h after removal (Supplementary Fig. S5A). This difference is likely attributable to differences in treatment concentrations (i.e. 2,500 µM IPTG vs. 0.1 µM estradiol) and the cell permeability of IPTG and estradiol. However, in contrast to their mRNA levels, Venus FL protein levels remained nearly constant throughout the 24 h after removal (Supplementary Fig. S5B), likely due to the high stability of the Venus protein (Chalfie et al. 1994). Thus, based on the observed changes in mRNA levels, the estradiol system may allow for the relatively rapid termination of induced gene transcription upon estradiol removal.

### Evaluation of possible side effects of IPTG and estradiol treatment, as well as LacI and ZFHD1 expression, on *C. merolae* growth and transcriptome

Finally, we investigated whether IPTG or estradiol treatment, or LacI and ZFHD expression, exhibit any side effects on *C. merolae* cells. To evaluate the possible effects of IPTG and estradiol, wild-type cultures were grown with 2,500 µM IPTG or distilled water as a vehicle control, and with 0.1 µM estradiol or acetone as a vehicle control, and the cellular growth rate (OD750 increase) was monitored over 8 d. As a result, no differences were observed between the vehicle control and IPTG- or estradiol-treated cultures, indicating that the working concentrations of IPTG and estradiol do not affect cellular growth (Supplementary Fig. S6A).

In addition, we evaluated the impact of IPTG and estradiol on the transcriptome in WT cells 6 h after the addition of 2,500 µM IPTG and 0.1 µM estradiol, a duration sufficient for the chemicals to exert their effects on target protein induction or degradation, as shown above. As a result, comparative transcriptome analyses based on RNA-seq showed no differentially expressed genes (DEGs, *FDR q*-value < 0.01, log_2_CPM > 2 and |log_2_FC| > 1) between the vehicle control and IPTG- or estradiol-treated cultures (Supplementary Fig. S6B; Supplementary Table S2). Thus, IPTG and estradiol impose negligible stress on *C. merolae* cells and are highly unlikely to affect their metabolism or physiology.

To evaluate the possible effects of LacI and ZFHD1 expression, we compared the growth and transcriptome profiles of the WT and *ZBS-cIL2p* (4*×lacO*)-*Venus FL* strains. The growth curves of the 2 strains were indistinguishable (Supplementary Fig. S6A). The growth curves of the 2 strains were indistinguishable (Supplementary Fig. S6A). However, transcriptome analysis identified 182 DEGs (138 upregulated and 44 downregulated) out of the 4,773 nucleus-encoded genes, excluding *ZFHD1 HA*, *LacI,* and *Venus FL,* which are expressed only in the *ZBS-cIL2p (4×lacO)-Venus FL* strains (Supplementary Fig. S6B, Supplementary Table S2). These DEGs potentially include those attributable to the parental background of the *ZBS-cIL2p* (4*×lacO*)-*Venus FL* strain—namely, strain M4, a spontaneous uracil-auxotrophic mutant (Minoda et al., 2004) that may carry mutations other than in the *URA5.3* locus—derived from the WT (strain 10D), rather than to the expression of LacI and ZFHD1-HA. Thus, to obtain a more accurate assessment, we referred to our previous RNA-seq dataset comparing the WT and the *mVenus^RD-SKP^* strain, which constitutively expresses mVenus-FRB and HA-FKBP-SKP1 and was also derived from M4 (Fujiwara et al. 2024). Among the 182 DEGs identified in the WT vs *ZBS-cIL2p* (4*×lacO*)-*Venus FL* comparison 64 genes (49 upregulated and 15 downregulated) were shared with those detected in the WT vs *mVenus^RD-SKP^* comparison. This overlap indicates that a subset of the DEGs reflects background differences between the WT and M4. In contrast, 118 DEGs (89 upregulated and 29 downregulated) were unique to the WT vs *ZBS-cIL2p* (4*×lacO*)-*Venus FL* comparison. Of these, 113 genes showed only minor expression changes (|log_2_FC| < 2), whereas only 5 genes—including 3 genes of unknown function (CMG127C, CMN038C, and CMR316C), 1 gene encoding an RNA-binding protein (CMH221C), and 1 gene encoding a hedgehog protein (CMO184C)—exhibited relatively large changes (downregulation; log_2_FC = −2.16, −2.27, −3.45, −2.49, −2.03, respectively) in the *ZBS-cIL2p (4×lacO)-Venus FL* strain (Supplementary Fig. S6B, Supplementary Table S2). Although the physiological significance of this downregulation remains unclear, the overall impact of ZFHD1-HA and LacI expression on global gene expression was limited. Moreover, IPTG-mediated release of LacI from the chromosome in the *ZBS-cIL2p* (4*×lacO*)-*Venus FL* strain had no detectable effects on either cellular growth or transcriptome profiles (Supplementary Fig. S6, Supplementary Table S2). Thus, no side effects of IPTG addition were observed in the background expressing ZFHD1 HA and LacI.

### Targeted protein knockdown of DRP5B by estradiol-induced NbALFA–SKP1

Previously, we successfully degraded the chloroplast division protein DRP5B (dynamin-related protein 5B), an endogenous essential gene product, using a rapamycin-dependent inducible protein knockdown system, which led to inhibition of chloroplast division (Fujiwara et al. 2024). This system also drives targeted protein degradation through the ubiquitin–proteasome pathway. In the present study, to evaluate the applicability of the nanobody-based inducible protein knockdown system, we again targeted DRP5B. The ALFA tag fused to mChartreuse (mC; a green fluorescent protein) was used as a degron–reporter module. This module was inserted immediately upstream of the start codon of the endogenous *DRP5B* locus in the pre-established estradiol-inducible NbALFA–SKP1 strain, generating the transformant *ALFA-mC-DRP5B^ESD::NbALFA-SKP1^* (Supplementary Fig. S7A). This strain enabled simultaneous monitoring of DRP5B localization and abundance together with its estradiol-inducible degradation.

When cultured asynchronously under continuous light, estradiol treatment inhibited the growth of *ALFA-mC-DRP5B^ESD::NbALFA-SKP1^* cells, ultimately leading to cell death, suggesting that DRP5B was effectively degraded and that depletion was sustained over time (Supplementary Fig. S7B). To further investigate chloroplast division defects, the culture was synchronized under a 12-h light/12-h dark cycle. Estradiol or vehicle (acetone) was added 4 h before the onset of the third light phase. Immunoblotting with an anti-GFP antibody revealed that, in vehicle-treated cells, ALFA–mC–DRP5B began to accumulate at hour 9 (the onset of the light period is defined as hour 0), reached a peak at hour 12, and then decreased, corresponding to the period of chloroplast division. In contrast, in estradiol-treated cells, NbALFA–SKP1 accumulated by hour 6, and ALFA–mC–DRP5B was nearly absent at hour 9 and only slightly expressed at hour 12, with levels substantially lower than in the vehicle control. Throughout the LD cycle, estradiol treatment reduced ALFA–mC–DRP5B protein levels to approximately 30% of the control (Supplementary Fig. S7C).

To confirm chloroplast division defects, we examined both the localization and abundance of ALFA–mC–DRP5B using fluorescence microscopy (Supplementary Fig. S7D). In vehicle control cells at hour 9, ALFA–mC–DRP5B fluorescence was undetectable. Although the protein was detectable by immunoblotting, it was likely not yet assembled into a ring and therefore below the threshold for microscopic detection. At hour 12, the protein localized to the chloroplast division site, as previously reported (Miyagishima et al. 2003), and chloroplast division was completed prior to telophase and cytokinesis at hour 16. In estradiol-treated cells, however, ALFA–mC–DRP5B fluorescence on the chloroplast was markedly reduced at hour 12, and chloroplast division was inhibited (Supplementary Fig. S7D). Consequently, between hours 16 and 24, cells initiated cytokinesis without undergoing chloroplast division (irregular cytokinesis; Supplementary Fig. S7D). Such cells accumulated only under estradiol treatment, reaching a maximum proportion of ∼40% at hour 16 (Supplementary Fig. S7E). This irregular cytokinesis represents a secondary consequence of chloroplast division defects in *C. merolae*, as previously reported (Sumiya et al. 2016; Fujiwara et al. 2024). Normally, duplicated nuclei and chloroplasts are distributed evenly to each daughter cell during cytokinesis (Supplementary Fig. S7F, left). In estradiol-treated cultures, however, cells containing a nucleus but no chloroplast were detected, indicating a failure of chloroplast partitioning (Supplementary Fig. S7F, right). Ultimately, chloroplast division defects led to cell death, and dead cells were observed as aggregates that had lost chloroplast fluorescence (Supplementary Fig. S7D, asterisks). Together, these results demonstrate that the inducible knockdown systems developed in this study provide a powerful approach for elucidating the functions of endogenous essential proteins in *C. merolae* by *conditional depletion*.

## Discussion

In this study, we successfully developed IPTG- and estradiol-inducible gene/protein expression systems in *C. merolae*. While practical IPTG-inducible systems have not been established in other eukaryotes due to issues such as leaky expression in the absence of IPTG and insufficient induction upon IPTG addition (Hu and Davidson 1987; Figge et al. 1988; Kjærulff and Nielsen 2015; Myung et al. 2020), we achieved precise control of gene expression in *C. merolae* by introducing 4 *lacO* sequences at appropriate positions within the *cIL2* promoter (Fig. 1; Supplementary Fig. S2). This modification enabled gene expression at the maximum activity of the promoter upon IPTG treatment while preventing leaky expression (Fig. 1). It is unclear whether this method would work effectively in other eukaryotes, but it is worth testing. The estradiol system is commonly used in land plants but has certain limitations due to some side effects (Upadhyay and Maier 2016; Adeel et al. 2017). On the other hand, although the reason remains unclear, similar to IPTG, it did not show any side effects in *C. merolae* (Supplementary Fig. S6), at least under standard culture conditions. In addition, the estradiol system yielded an expression induction comparable to that of the IPTG system (1.5 to 1.7 fold; Fig. 4F). Given these features, these gene induction systems, together with the protein knockdown systems developed here through their modification, will be especially useful for examining cellular metabolism, physiology, and responses to environmental changes, since neither IPTG nor estradiol itself affects these processes. In addition, it should be noted that in the IPTG system, the level of protein induction is adjustable by varying the IPTG concentration (Fig. 1).

Regarding the protein knockdown systems, we demonstrated the inducible degradation of cytosolic Venus protein by nanobody-conjugated SKP1, a component of the SCF E3 ubiquitin ligase complex, upon IPTG or estradiol treatment as a proof of concept (Figs. 2, 3, and 5). In addition, we confirmed the utility of these inducible systems by successfully degrading the endogenous chloroplast division protein DRP5B, which was tagged with the ALFA degron tag and mChartreuse, upon inducible expression of NbALFA–SKP1 (Supplementary Fig. S7). Since we previously succeeded in degrading *C. merolae* endogenous proteins conjugated with the FRB tag, such as the nuclear-localized protein E2F, upon rapamycin treatment using FKBP-conjugated SKP1 and observed the resultant phenotypes (Fujiwara et al. 2024), the IPTG and estradiol systems should also be applicable to the analysis of such nuclear proteins. Furthermore, the effect of rapamycin lasted only 4 to 8 h, requiring repeated administration to maintain long-term depletion of the target protein, and it exhibited slight side effects on cells (Fujiwara et al. 2024). In contrast, the effects of IPTG and estradiol persisted for at least 72 h with a single dose (Figs. 1 to 5) and showed no side effects (Supplementary Fig. S6), making them more effective than the rapamycin-dependent system.

Regarding the kinetics of the IPTG- or estradiol-induced system, it took 6 or 4 h after reagent addition for more than 80% of the target protein (Venus protein) to be degraded (Figs. 2, 3, and 5). Because Venus is highly stable (Chalfie et al. 1994), endogenous proteins with shorter lifetimes are likely to be eliminated more quickly by these systems. Still, a duration of 4 to 6 h falls within a sufficiently usable range considering the growth rate of *C. merolae*. As in many other unicellular eukaryotic algae, in the synchronous culture of *C. merolae* under a 12-h light/12-h dark cycle, the G1 phase of the cell cycle lasts approximately 12 h during the light period, followed by the S and M phases during the dark period, which take approximately 8 h (Miyagishima et al. 2012, 2014; Fujiwara et al. 2020).

Among the red algal class Cyanidiophyceae, *Galdieria* has also recently become genetically tractable by applying a method that essentially relies on the same homologous recombination approach as in *C. merolae* (Hirooka et al. 2022). Unlike the obligatory photoautotrophic *C. merolae* and other members of Cyanidiophyceae such as *Cyanidiococcus* and *Cyanidium*, *Galdieria* is capable of heterotrophic growth in the dark and mixotrophic growth in the light by assimilating a wide variety of sugars and sugar alcohols (Rigano et al. 1977; Oesterhelt et al. 1999; Oesterhelt and Gross 2002; Barbier et al. 2005). Additionally, under heterotrophic conditions, it exhibits plasticity, such as reversibly bleaching its plastid (Gross and Schnarrenberger 1995; Schönknecht et al. 2013). Furthermore, sexual reproduction, which remains unknown in other members of Cyanidiophyceae, has been discovered in *Galdieria*. (Hirooka et al. 2022). Thus, introducing the methods developed in this study to Galdieria would enable the analysis of phenomena that are not investigable in *C. merolae*.

Regarding further improvements to the methods, the IPTG system in *C. merolae* could be simplified. In this study, we used the *ZBS-cIL2* chimeric promoter because the locations of functional elements such as the TATA box and transcription start site within *cIL2* were known, as this information was important for designing the insertion sites of *lacO* sequences (Fig. 1; Supplementary Fig. S2). However, such information was not yet available for any endogenous promoters in the *C. merolae* genome. Since *cIL2* alone is unable to initiate transcription, *ZBS* sequence was added, and ZFHD1, which binds to it, was also expressed. Nevertheless, in principle, an endogenous promoter with constitutive strong transcriptional activity in *C. merolae* should be usable instead of *ZBS-cIL2* promoter. In that case, there would be no need to introduce a cassette for expressing ZFHD1.

Since IPTG- and estradiol-systems function independently through different mechanisms, they can be used, for example, to sequentially induce or degrade 2 different proteins or 2 groups of proteins at different time points. In the latter case, one group of proteins conjugated with the same epitope recognized by a nanobody will be simultaneously degraded upon the addition of IPTG or estradiol. Regarding nanobodies, in addition to the GFP and ALFA tag nanobodies used in this study, small epitope tags such as BC2 (Virant et al. 2018) and Pep (Traenkle et al. 2020), along with their corresponding nanobodies, could also be utilized. Such stepwise experiments would be useful for analyzing mechanisms in which individual proteins or groups of proteins are expressed and function sequentially, such as in the cell cycle and circadian rhythms.

Until recently, an effective inducible protein knockdown system had not been available for land plants. However, a method called E3-Targeted Degradation of Plant Proteins (E3-DART), which is induced by dexamethasone, was developed (Huang and Rojas-Pierce 2024). The E3-DART system repurposes the pathway in which *Salmonella*, after infecting a host human cell, uses its own SspH1 protein, which acts as an E3 ubiquitin ligase, to bind to human protein kinase 1 (PKN1), leading to the degradation of PKN1 by the host proteasome. In this process, the LRR-NEL domain of *Salmonella* SspH1 binds to the HR1b domain of human PKN1 (Keszei et al. 2014; Cook et al. 2019). In the E3-DART system in plants, an HR1b-fused target protein is ubiquitinated by the LRR-NEL domain, which is expressed upon DEX treatment, and subsequently degraded by the proteasome (Huang and Rojas-Pierce 2024). Thus, the E3-DART system differs mechanistically from, and should act independently of, the method used in this study, even if they coexist, as the latter relies on nanobody-mediated recognition of target proteins. Therefore, testing the combination of the E3-DART system with the nanobody system in *C. merolae* in future studies would be valuable.

As a future perspective, another potential application of the inducible systems developed in this study may be their use in establishing RNAi in Cyanidiophyceae, which naturally lack this machinery. Inducible RNAi systems have already been established in various organisms, including cultured animal cells and land plants (Wiznerowicz and Trono 2003; Wielopolska et al. 2005; Flores-Sandoval et al. 2016). In this regard, in the budding yeast *Saccharomyces cerevisiae*, which also evolutionarily lost the RNAi machinery, the introduction of 2 core RNAi genes, *Dcr1* and *Ago1*, from the closely related yeast *S. castellii* (reclassified as *Naumovozyma castellii*; Kurtzman and Robnett 2003; Karademir Andersson and Cohn 2017) was sufficient to reconstitute the pathway (Drinnenberg et al. 2009). By analogy, a similar approach may also be applicable to *C. merolae*. Such an inducible RNAi system would provide a complementary strategy in cases where protein-based knockdown is inefficient. In such cases, combining mRNA- and protein-targeting approaches could enable more complete elimination of the target. In addition, RNAi has the advantage of not requiring tagging of the endogenous protein, thereby broadening its applicability.

In summary, the IPTG- and estradiol-inducible gene and protein expression systems established in this study enable both gain- and loss-of-function analyses with minimal side effects. Their potential applicability to other genetically tractable members of Cyanidiophyceae (Hirooka et al. 2022) and diverse unicellular algae highlights their value as a platform for comparative and evolutionary studies.

## Materials and methods

### Culture conditions of *C. merolae*

*C. merolae* 10D wild type (NIES-3377), the uracil-auxotrophic mutant M4 (a derivative of *C. merolae* 10D, which has a lethal mutation in the *URA* gene; Minoda et al., 2004), and its transformants were maintained in MA2 medium (an inorganic medium; Ohnuma et al., 2008). For the M4 strain, the medium was supplemented with 0.5 mg mL^−1^ uracil. All transformant strains generated in this study were maintained in 20 mL of the medium in 25-cm^2^ tissue culture flasks (90026; TPP Techno Plastic Products AG) in the light (30 µmol m^−2^ s^−1^) at 42 °C in an incubator shaker (BR-43FL; TITEC, Japan) with agitation at 120 rpm.

### Preparation of *C. merolae* transformants

We first constructed plasmids containing gene expression cassettes and the *URA* selection marker flanked by upstream and downstream homologous arms for homologous recombination-mediated genetic modification. The constructs were then amplified by PCR, and the resulting linear DNA was introduced into *C. merolae* M4 using a polyethylene glycol (PEG)-mediated transformation method (Ohnuma et al., 2008). For plasmid construction, the DNA sequences for the constructs were either amplified from *C. merolae* genomic DNA by PCR or artificially synthesized and assembled using the In-Fusion Snap Assembly Master Mix (Takara Bio, Japan). The plasmid sequences are shown in Supplementary Table S1. The DNA constructs for *C. merolae* transformation were amplified from the corresponding plasmids by PCR with the primer set (forward 5′-acaatttcacacaggaaacagctatgac-3′ and reverse 5′-cgttgtaaaacgacggccagt-3′) and then purified using a NucleoSpin Gel and PCR Clean-up (Takara). All constructs except for *ALFA–mC–DRP5B*, were integrated into the upstream region of the chromosomal *URA* locus in the *C. merolae* M4 strain, using 2 to 4 µg of purified DNA for transformation.

To generate the *ALFA–mC–DRP5B^ESD::NbALFA-SKP1^* strain, we used a construct carrying the nucleotide sequence encoding ALFA tag and the green fluorescent protein mChartreuse fused to *DRP5B*, together with the blasticidin deaminase (*BSD*) selection marker (Fujiwara et al. 2021) and homologous arms for targeting the *DRP5B* locus. This construct was introduced into the estradiol-inducible *NbALFA–SKP1* background strain, which had been generated separately by *URA* locus integration in the wild-type 10D strain with the chloramphenicol acetyltransferase (*CAT*) selection marker (Fujiwara et al. 2017).

### Antibody generation

To generate antibodies against *C. merolae* SKP1 and CUL1, the N-terminal 150 amino acids of SKP1 (CMP118C) and the C-terminal 300 amino acids of CUL1 (CMT046C), each tagged with a 6×His sequence, were expressed in *E. coli*, purified, and used to immunize rabbits. The resulting antisera were then affinity-purified using the corresponding recombinant peptides as ligands.

### IPTG and estradiol treatments and immunoblotting

All strains were initially diluted to an OD750 of 0.2 in 20 mL of MA2 medium in 25-cm^2^ tissue culture flasks and cultured with agitation (150 rpm) at 42 °C under continuous light (70 μmol m⁻^2^ s⁻^1^) for 2 d. Then IPTG or estradiol was added to the cultures.

For the IPTG treatment, stock solutions of 10, 50, 100, 250, 500, and 1,000 mm IPTG (FUJIFILM Wako Pure Chemical) were prepared in distilled water. To achieve the final concentrations of 0, 50, 100, 250, 500, and 1000 μM in cultures, the corresponding stock solutions were added at a 1:1,000 dilution. Higher concentrations of 2,500 and 5,000 μM were obtained by diluting the 1,000 mm stock solution 1:400 and 1:200, respectively.

For the estradiol treatment, stock solutions of 0.001, 0.005, 0.01, 0.05, 0.1, and 1 mm estradiol (FUJIFILM Wako Pure Chemical) were prepared in acetone. To obtain the final concentrations of 0.001 μM, 0.005, 0.01, 0.05, 0.1, and 1 μM in cultures, these stock solutions were added at a 1:1,000 dilution.

After the addition of IPTG or estradiol, 1 mL of *C. merolae* culture was harvested at specific time points by centrifugation at 6,000 × *g* for 1 min at room temperature. The cell pellets were lysed with the sample buffer (2% [w/v] SDS, 62 mm Tris-HCl, pH 6.8, 100 mm DTT, 10% [w/v] glycerol, and 0.01% [w/v] bromophenol blue) and then incubated at 95 °C for 5 min. After centrifugation at 20,000 × *g* for 5 min, the protein concentration in the supernatant was measured using an XL-Bradford kit (Aproscience). The whole cell extracts containing total proteins (6 µg) were separated on polyacrylamide gels by SDS–PAGE and then transferred to PVDF membranes (Immobilon, Millipore). The membranes were blocked with 5% skim milk dissolved in TTBS (20 mm Tris-HCl, pH 7.5, 200 mm NaCl, 0.1% [v/v] Tween 20). The primary antibodies were diluted in Bullet ImmunoReaction Buffer (Nacalai Tesque) and used at the following dilutions: anti-GFP (for detection of Venus; dilution of 1:2,000; clone JL-8, Takara), anti-HA (1:5,000; clone 16B12, BioLegend), anti-V5 (1:3,000; clone OZA3, MBL), anti-SKP1 (250 ng/mL) and anti-CUL1 (250 ng/mL). As secondary antibodies, HRP-conjugated antimouse or antirabbit IgG (1:40,000; Thermo Fisher Scientific) was used. The signals were detected using SuperSignal West Atto Ultimate Sensitivity Substrate (Thermo Fisher Scientific) and ChemiDoc Touch Imaging System (BIO-RAD). The signal intensities of target proteins were quantified using ImageJ (https://imagej.net/ij/index.html). Biological triplicates of immunoblotting were performed to determine the change in the relative signal intensity and Sd of Venus, Venus FL, NbGFP-SKP1, and NbALFA-SKP1 proteins. Rodbard method in ImageJ and Excel (Microsoft) were used to generate the fitting curves.

### Microscopy

Differential interference contrast (DIC) and fluorescent images were captured using a fluorescence microscope (BX51, Olympus) equipped with a digital CCD camera system (DP71, Olympus). The following filter sets were used: LF514-C-U03 (Semrock) for Venus fluorescence, U-MNIBA3 (Olympus) for mChartreuse fluorescence, and U-MWIG3 (Olympus) for chloroplast autofluorescence.

### Growth curve

To monitor the growth of wild-type cultures with or without IPTG or estradiol, and of *ZBS-cIL2p (4×lacO)–Venus FL* cultures with or without IPTG, all cultures were initially diluted to an OD_750_ of 0.2 in 20 mL of MA2 medium and incubated in 25-cm^2^ tissue culture flasks with agitation (150 rpm) at 42 °C under continuous light (70 μmol photons m^−2^ s^−1^) for 2 d. After this preculture, the culture was again diluted again to an OD_750_ of 0.2 in 20 mL of fresh MA2 medium. IPTG or estradiol was then added to the cultures at final concentrations of 2,500 or 0.1 µM, respectively. The IPTG stock solution, prepared at 1,000 mm in distilled water, was added at a 1:400 dilution, while the estradiol stock solution, prepared at 0.2 mm in acetone, was added at a 1:2,000 dilution. Distilled water or acetone was added to control cultures as vehicle treatments. Biological triplicates were conducted to compare the growth rates of the cultures under each condition.

### Transcriptome analysis

Wild-type cells were precultured for 2 d, diluted, and then treated for 6 h with 2,500 µM IPTG, 0.1 µM estradiol, or vehicle only, under the same culture conditions described above. Similarly, *ZBS-cIL2p (4×lacO)–Venus FL* cells were treated for 6 h with 2,500 µM IPTG or vehicle only. In both cases, the cells were harvested by centrifugation at 2,000 × *g* for 5 min at 4 °C, rapidly frozen in liquid nitrogen, and stored at −80 °C until further use. Total RNA was extracted following the Trizol/RNeasy hybrid protocol (Trizol, Life Technologies; RNeasy Mini Kit, Qiagen) according to the manufacturer's instructions. To construct a cDNA library of 150 bp, 100 ng of total RNA were used. Paired-end sequencing was performed using the Illumina sequencing platform (Novaseq 6000) according to the manufacturer's instructions (Illumina). The coding sequence (CDS) was used as a reference for the *C. merolae* transcripts. To obtain gene expression scores, one side of the trimmed paired-end reads was mapped to the reference by Bowtie2 ver. 2.3.4.1 (Langmead and Salzberg 2012). SAMtools ver. 1.8 (Li et al. 2009), BEDtools ver. 2.19.1 (Quinlan and Hall 2010), and R ver. 3.5.3 (Ihaka and Gentleman 1996) were used to calculate the number of reads mapped to the contigs (raw count). To detect differences in the transcriptomes, count data from 4 biological replicates were analyzed using edgeR ver. 4.4.0 (Robinson et al. 2010) in R. Genes were identified as DEGs only when FDR was <0.01, log_2_CPM was >2 and the absolute value of log_2_FC was >1. The MA plots were generated using the normalized count data.

In addition, raw count data obtained in our previous study (Fujiwara et al. 2024) comparing the WT and *mVenus^RD-SKP1^* strain were re-analyzed using the same edgeR pipeline to identify DEGs. For consistency across datasets, the count values were converted to TPM (transcripts per million). The results were compiled together with the present transcriptome analysis and were provided in Supplementary Table S2.

### RT-qPCR analysis of *Venus FL* mRNA levels following the removal of IPTG or estradiol

The *ZBS*-*cIL2p* (4*×lacO*)-*Venus FL* and *ESD::Venus FL* cultures were initially diluted to an OD750 of 0.2 in 20 mL of MA2 medium in 25-cm^2^ tissue culture flasks and incubated with agitation (150 rpm) at 42 °C under continuous light (70 μmol m⁻^2^ s⁻^1^) for 2 d. After that, IPTG or estradiol was added to the respective culture at a final concentration of 2,500 μM IPTG or 0.1 μM estradiol. Twenty four hours after the addition, the cells were harvested by centrifugation at 2,000 × *g* for 5 min at 4 °C, rapidly frozen in liquid nitrogen, and stored at −80 °C until further analysis. Total RNA was extracted using the Trizol/RNeasy hybrid protocol. Subsequently, 1 µg of total RNA was reverse-transcribed into cDNA using PrimeScript reverse transcriptase (Takara) with oligo(dT) primers, following the manufacturer's instructions. Q-PCR was performed using the CFX Duet Real-Time PCR System (Bio-Rad) and SsoAdvanced Universal SYBR Green Supermix (Bio-Rad), with primer sets for *Venus FL* (forward: 5′-CAGAACACCCCAATCGGTGA-3′, reverse: 5′-GTTCGGGTCCTTCGAGAGTG-3′) and dynamin-related protein 3 (*DRP3*) (forward: 5′-CGCGAATAGTGCACCGAAAG-3′, reverse: 5′-GCATCGTCGTTCTTCTTCGC-3′), using cDNA as the template. The *Venus FL* mRNA levels were normalized to those of *DRP3*, which is constitutively expressed and serves as an internal control (Fujiwara et al. 2015).

### Comparative RT–qPCR analysis of Venus FL mRNA levels among endogenous promoter–driven and inducible strains

Cultures (20 mL) of endogenous promoter–driven *Venus FL* strains (*CATAp, FBA2p, CABp, CPCCp, K024p*) and inducible strains (*ZBS-cIL2p* (*4×lacO*) and *ESD::Venus FL*) were inoculated to an OD_750_ of ∼0.2 and grown in 25-cm^2^ tissue culture flasks with agitation (150 rpm) at 42 °C under continuous light (70 µmol m^−2^ s^−1^) for 3 d. Inducing reagents (2,500 µM IPTG with distilled water as the vehicle, or 0.1 µM estradiol with acetone as the vehicle) were added at the start of the culture. Cultures were harvested on day 3. Total RNA extraction, cDNA synthesis, and RT-qPCR analysis were performed as described above. Relative transcript levels were quantified using the standard curve method, with *Venus FL* expression normalized to *DRP3*. Expression levels were further normalized to those driven by the *CATA* promoter, which was set to 100%.

### Accession numbers

Amino acid sequence data from this article can be found in the following public databases: SKP1 (*C. merolae* Genome Project http://czon.jp, CMP118C), CUL1 (CMT046C), DRP5B (CMN262C), APCC (CMO250C), CPCC (CMP166C), CAB (CMN234C), FBA2 (CMI049C), Catalase (CMI050C); Venus (fpbase.org/protein/venus/), mChartreuse (fpbase.org/protein/mchartreuse/); NbGFP (PDB ID 3OGO), NbALFA (PDB ID 6I2G). The nucleotide sequences in the constructs used to produce transformant strains in this study are shown in Supplementary Table S1.

## Supplementary Material

kiaf575_Supplementary_Data

## Data Availability

The RNA-seq data obtained in this study have been deposited in the NCBI Sequence Read Archive (SRA) under BioProject ID PRJNA1228989. The corresponding BioSample accession numbers are SAMN47118521–SAMN47118536, SAMN51764534–SAMN51764537, and SAMN51764542–SAMN51764549. All other relevant data are included in the manuscript and/or provided as supporting information.

## Supplementary data

The following materials are available in the online version of this article.

**Supplementary Figure S1.**  *cIL2p* functions with the artificial transcription factor ZFHD1 and its binding sequences in *C. merolae*

**Supplementary Figure S2.** The DNA sequence of *ZBS* and *cIL2p*, as well as the positions of the *lacO* insertions

**Supplementary Figure S3.** Comparison of the efficiency of IPTG-inducible protein knockdown mediated by NbGFP-SKP1 and NbGFP-CUL1

**Supplementary Figure S4.** The DNA sequence of the *LBS*

**Supplementary Figure S5.** Changes in Venus FL mRNA and protein levels following the removal of IPTG and estradiol in the *ZBS-cIL2p* (4*×lacO*) and *ESD::Venus FL* strains, respectively

**Supplementary Figure S6.** Growth and transcriptome analyses of wild-type (WT) *C. merolae* treated with IPTG or estradiol, comparison between WT and *ZBS-cIL2p* (4*×lacO*), and effects of IPTG treatment on *ZBS-cIL2p* (4*×lacO*)

**Supplementary Figure S7.** Targeted protein knockdown of DRP5B by estradiol-induced NbALFA-SKP1

**Supplementary Table S1.** DNA sequences of the constructs used to generate the transformant strains in this study.

**Supplementary Table S2.** RNA-Seq transcriptome datasets generated in this study.

## Lead contact

Further information and requests for resources and reagents should be directed to and will be fulfilled by the lead contact, Takayuki Fujiwara (tkfujiwara@nig.ac.jp).

## Materials availability

All plasmids and strains generated in this study are available to the lead contact upon request.

## Code availability

This study did not generate any code.

## Dive Curated Terms

The following phenotypic, genotypic, and functional terms are of significance to the work described in this paper:

ZFHD1 Gramene: AT1G69600
ZFHD1 Araport: AT1G69600

AGO1 Gramene: AT1G48410
AGO1 Araport: AT1G48410

GFP Gramene: green fluorescent protein
GFP Araport: green fluorescent protein
