# Supplementary material for: IPTG- and estradiol-inducible gene expression systems in the unicellular red alga Cyanidioschyzon merolae
Source: Plant Physiol. 2025 Nov 10;200(1):kiaf575. doi: 10.1093/plphys/kiaf575 (PMC12770821; doi:10.1093/plphys/kiaf575)
Supplement: kiaf575_Supplementary_Data [file kiaf575_supplementary_data.zip › PLPHYS-2025-0665R1-Supplementary Figures with Legends.pdf]

Supplementary Figure S1

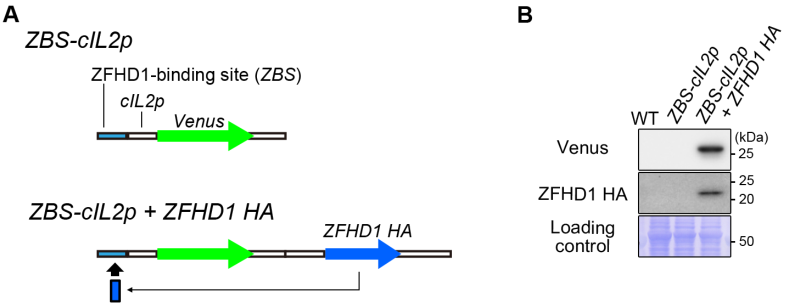

Supplementary Figure S1. *cIL2p* functions with the artificial transcription factor ZFHD1 and its binding sequences in *C. merolae*

(A) Schematic illustration of the constructs used to generate the *ZBS-cIL2p* and *ZBS-cIL2p + ZFHD1 HA* strains. The first line illustrates the construct used to generate the *ZBS-cIL2p* strain, which contains the ZFHD1-binding site (*ZBS*; 12× ZFHD1-binding sequences), followed by *cIL2p* for RNA polymerase II binding, the *Venus* open reading frame (ORF), and the *UBIQUITIN* 3′ downstream sequence (*UBQ*-3'utr). The second line illustrates the construct used to generate the *ZBS-cIL2p + ZFHD1 HA* strain, which additionally contains an expression cassette for ZFHD1 tagged with 3×HA tags (ZFHD1 HA). This cassette consists of the constitutive *CMK024C* promoter, the *ZFHD1 HA* ORF, and the *APX* 3′ downstream sequence (*APX*-3'utr). The constructs were integrated into the upstream region of the *URA* locus (a chromosomal neutral site). The detailed sequences are shown in Supplementary Figure S2 and Supplementary Table S1.(B) Immunoblotting of Venus (detected with the anti-GFP antibody) and ZFHD1 HA (detected with the anti-HA antibody) in the wild-type (WT) strain as a control, the *ZBS-cIL2p* strain, and the *ZBS-cIL2p-Venus-ZFHD1 HA* strain.

Supplementary Figure S2

A ZFHD1-binding site (*ZBS*, 12 copies of ZFHD1-binding sequece)

single ZFHD1-binding sequence

TAATGATGGGCG

accgcggcttacgcgtgctagc**TAATGATGGGCG**ctcgag**TAATGATGGGCG**gtcgac**TA**

ATGATGGGCGctcgag**TAATGATGGGCG**tctagc**TAATGATGGGCG**ctcgag**TAATGATG**

GGCGgtcgac**TAATGATGGGCG**ctcgag**TAATGATGGGCG**tctagc**TAATGATGGGCG**ct

cgag**TAATGATGGGCG**gtcgac**TAATGATGGGCG**ctcgag**TAATGATGGGCG**

B *Interleukin-2* core promoter (*cIL2p*)

▲ ***lacO* operator**

TTGTGAGCGGATAACAA

cIL2p

tctagaacgcgaattaattcaa**ATTTTGACACCCCCATAATATTTTTCCAGAATTAACAG**

▲4th

TATA box — Initiator

TATAAATTGCATCTCTTGTTCAAGAGTTCCCTATCACTCTCTTTAATCACTACTCACAGT

▲1st ▲2nd

start codon

AACCTCAACTCCTGCcacaagcttgccctgcagcgggaattcc**ATG**...

▲3rd

Supplementary Figure S2. The DNA sequences of ZFHD1-binding site (***ZBS***) **and *cIL2p*, as well as the positions of the *lacO* insertions**

(A) The *ZBS* consists of 12 copies of the ZFHD1-binding sequences. A single ZFHD1-binding sequence is underlined in blue. (B) The *cIL2p* sequence and its flanking regions are shown. *cIL2p* is highlighted with a box. Functional elements, such as the TATA box and initiator, are indicated in underlined red text. Purple arrowheads mark the positions where *lacO* operator sequences were inserted.

Supplementary Figure S3

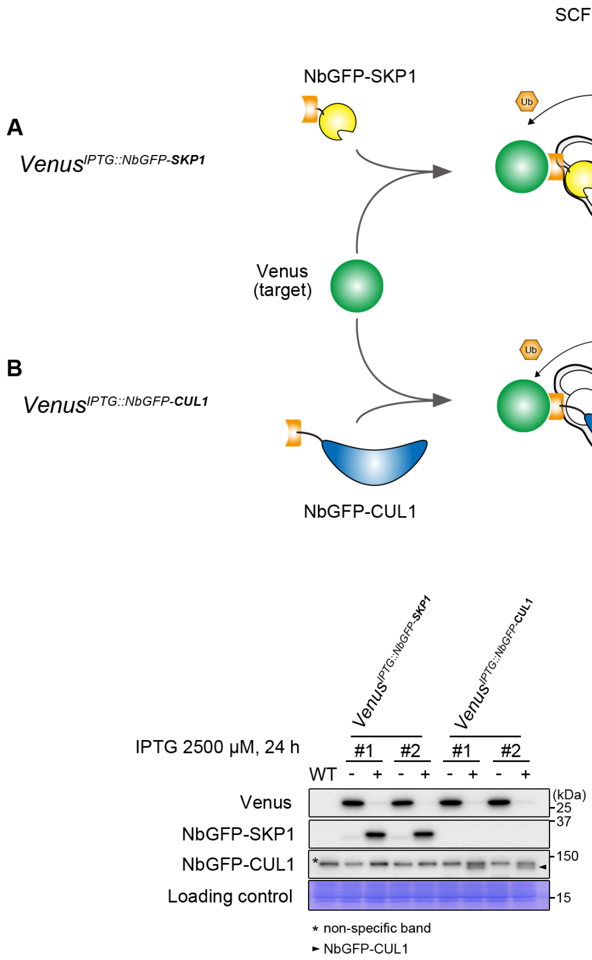

Supplementary Figure S3. Comparison of the efficiency of IPTG-inducible protein knockdown mediated by NbGFP-SKP1 and NbGFP-CUL1

(A) Schematic representation of the systems. The *Venus*$^{IPTG::NbGFP\text{-}SKP1}$ and *Venus*$^{IPTG::NbGFP\text{-}CUL1}$ strains strains were generated to constitutively express Venus as a degradation target under the control of the *EFTU* promoter and to inducibly express the anti-GFP nanobody conjugated with either SKP1 (NbGFP-SKP1) or CUL1 (NbGFP-CUL1) as targeted ubiquitination inducers under the control of the *ZBS-cIL2p* (4×*lacO*) promoter upon IPTG treatment. These proteins were expressed from the upstream region of the *URA* locus (a chromosomal neutral site). The detailed sequences are shown in Supplementary Figure S2 and Supplementary Table S1. Both SKP1 and CUL1, components of the SCF E3 ligase, were previously utilized in rapamycin-inducible protein knockdown systems developed in *C. merolae* (Fujiwara et al., 2024). NbGFP-SKP1 and NbGFP-CUL1 proteins bind to the target protein Venus, leading to its ubiquitination by the SCF E3 ligase, which ultimately directs Venus for degradation through the proteasome. (B) Comparison of degradation efficiency. The *Venus*$^{IPTG::NbGFP\text{-}SKP1}$ and *Venus*$^{IPTG::NbGFP\text{-}CUL1}$ cultures were treated with 2,500 µM IPTG for 24 h. Immunoblotting showing the effect of IPTG on the expression of NbGFP-SKP1 (32 kDa; detected with the anti-SKP1 antibody) and NbGFP-CUL1 (128 kDa; detected with the anti-CUL1 antibody), as well as the degradation of Venus (27 kDa; detected with the anti-GFP antibody). The wild-type (WT) served as a control. The Coomassie Brilliant Blue (CBB) stained PVDF membrane is shown as a loading control.

Supplementary Figure S4

LexA-binding site (*LBS*, 4 copies of LexA operator)

single LexA operator

CTGT------ACAG

ggggaattgatccccccctcgacagcttgcatgccagcttgggctgcaggtcgaggctaaa

aaactaatcgcattatcatcccctcgacgta**CTGT**ACATATAACCACTGGTTTTATAT**AC**

AGcagta**CTGT**ACATATAACCACTGGTTTTATAT**ACAG**cagtcgacgta**CTGT**ACATATA

ACCACTGGTTTTATAT**ACAG**cagta**CTGT**ACATATAACCACTGGTTTTATAT**ACAG**cagt

cgaggtaagattagatatggatatgtatatggatatgtatatggtggtaatgccatgtaa

cIL2p

tatgctcgactctaggatcttctctag....

Supplementary Figure S4. The DNA sequence of the LexA-binding site (*LBS*)

The *LBS* consists of 4 copies of the LexA operator. LexA operator sequences are underlined in magenta.

Supplementary Figure S5

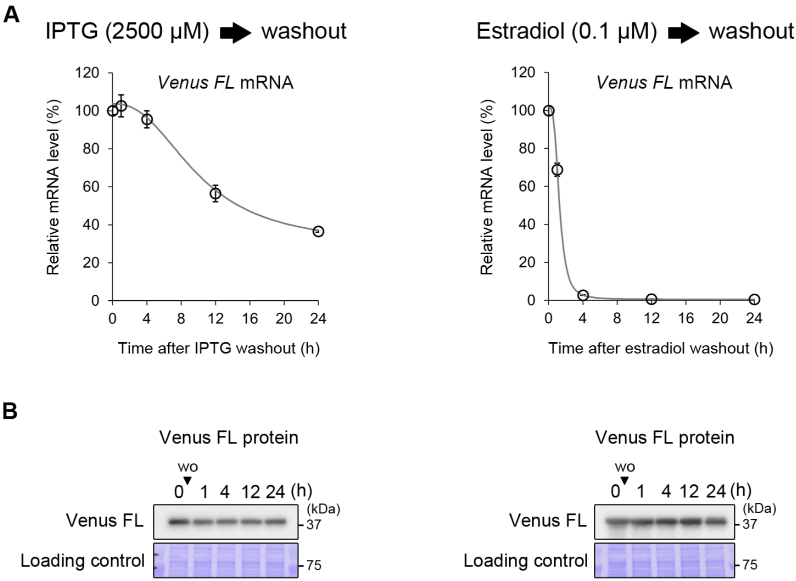

Supplementary Figure S5. Changes in Venus FL mRNA and protein levels following the removal of IPTG and estradiol in the *ZBS-cIL2p* (4×lacO) and *ESD::Venus FL* strains, respectively

The *ZBS-cIL2p* (4×*lacO*) and *ESD::Venus FL* cultures cultures were treated with 2,500 µM IPTG and 0.1 µM estradiol, respectively, for 24 h. After collecting the samples at 0 h, each reagent was washed out by centrifugation and resuspended in fresh media without IPTG or estradiol, and the cultures were further incubated. The cultures were subsequently collected at 1, 4, 12, and 24 h after washing for RT-qPCR and immunoblot analyses. (A) RT-qPCR analysis of *Venus FL* mRNA levels. The mRNA level at 0 h was defined as 100% in each strain. The plots and error bars represent the averages and standard deviations of three technical replicates. The half-life ($t_{1/2}$) was calculated using curve fitting in Excel. (B) Immunoblotting with the anti-GFP antibody showing Venus FL (37 kDa) levels. The Coomassie Brilliant Blue (CBB) -stained PVDF membranes are shown as loading controls.

Supplementary Figure S6

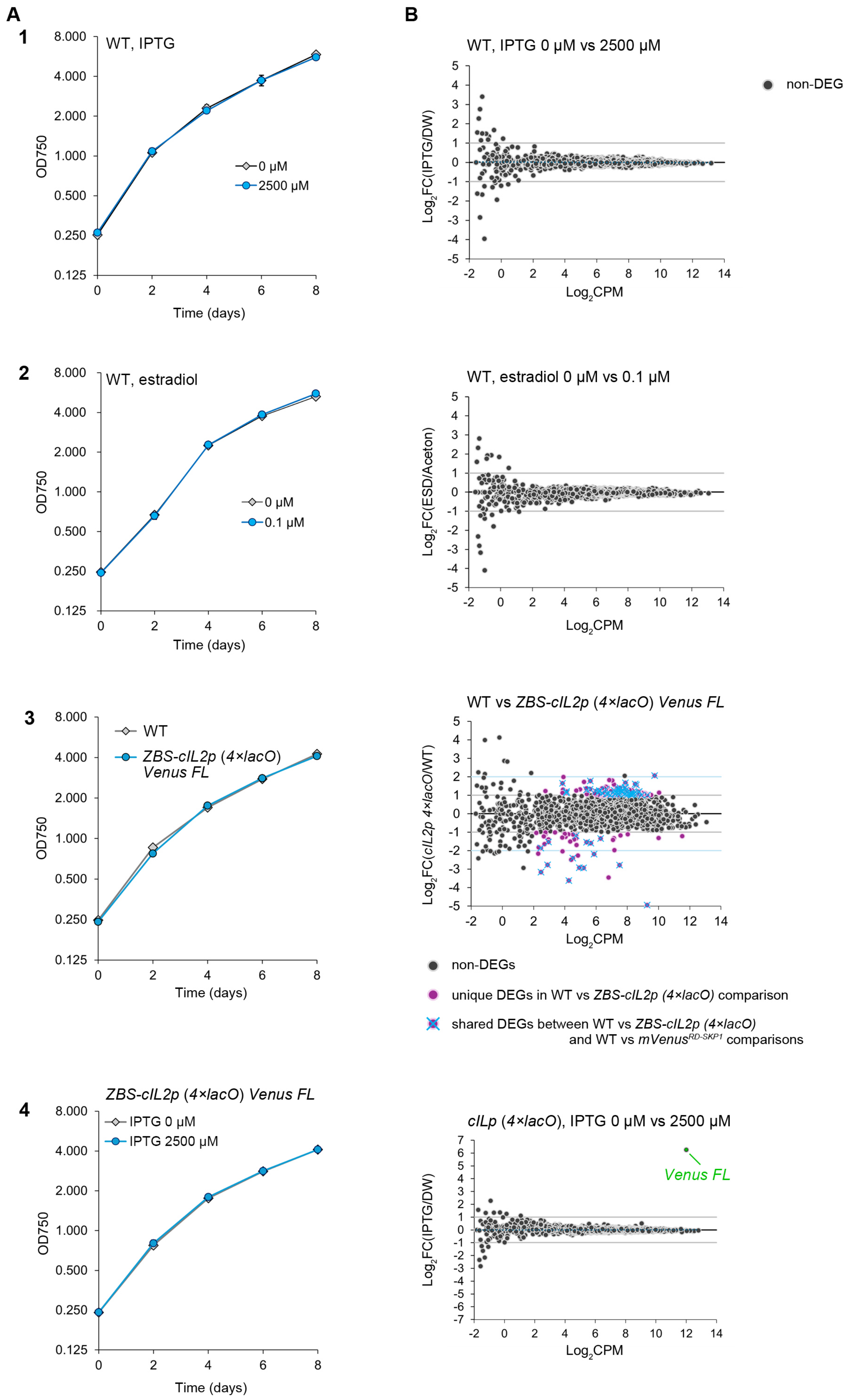

Supplementary Figure S6. Growth and transcriptome analyses of wild-type (WT) *C. merolae* treated with IPTG or estradiol, comparison between WT and *ZBS-cIL2p* (4×*lacO*)-*Venus FL*, and effects of IPTG treatment on *ZBS-cIL2p* (4×*lacO*)-*Venus FL*

(A) Growth was monitored by measuring the optical density at 750 nm (OD750). The plots and error bars represent the mean ± standard deviation of three biological replicates. From top to bottom: (1) WT treated with IPTG, (2) WT treated with estradiol, (3) comparison between WT and *ZBS-cIL2p* (4×*lacO*)-*Venus FL*, and (4) effects of IPTG treatment on *ZBS-cIL2p* (4×*lacO*)-*Venus FL*. (B) MA plots of RNA-seq data, presented in the same order as in (A). Gray dots indicate non–differentially expressed genes (non-DEGs), magenta dots indicate DEGs unique to the WT vs. *ZBS-cIL2p* (4×*lacO*)-*Venus FL* comparison, and magenta dots with blue crosses indicate DEGs shared between the WT vs *ZBS-cIL2p* (4×*lacO*)-*Venus FL* and WT vs $mVenus^{RD\text{-}SKP1}$ comparisons (Fujiwara et al., 2024). The mRNA expression levels (transcripts per million [TPM]), $\log_2$ fold change ($\log_2$FC), $\log_2$ counts per million ($\log_2$CPM), and false discovery rate q-values (FDR q-values) for each gene are provided in Supplementary Table S2. DEGs were defined by the following criteria: FDR q-value < 0.01, $\log_2$CPM > 2, and $|\log_2\text{FC}| > 1$.

Supplementary Figure S7

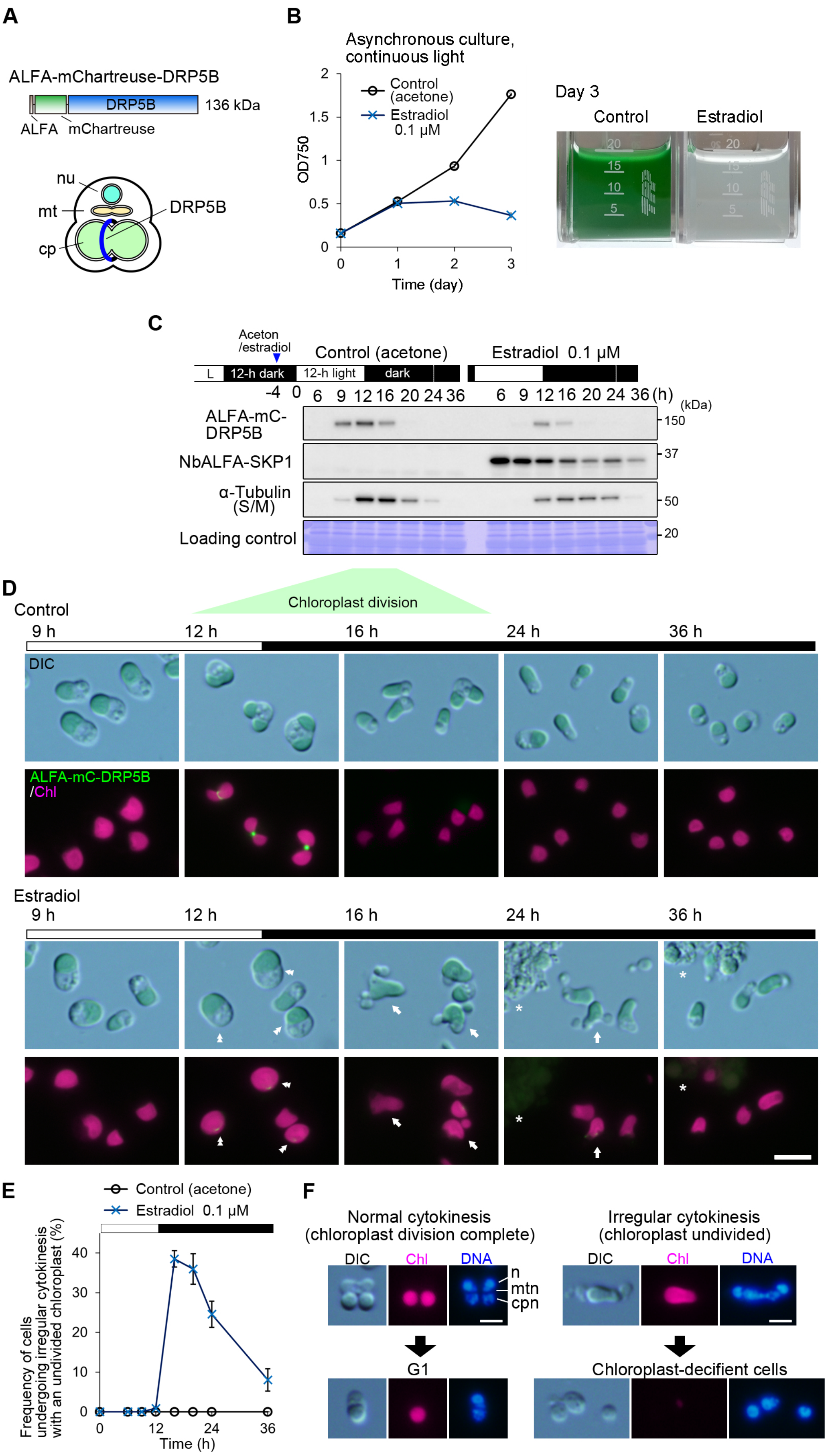

Supplementary Figure S7. Targeted protein knockdown of DRP5B by estradiol-induced NbALFA-SKP1

(A) Schematic representation of the ALFA-mChartreuse (mC) -DRP5B construct integrated into the endogenous DRP5B locus in the NbALFA-SKP1 inducible strain. The ALFA degron tag and mChartreuse green fluorescent protein were fused to the amino terminus of the chloroplast division protein DRP5B, which localizes on the cytosolic side of the chloroplast division site (Miyagishima et al., 2003). *n*, nucleus; *mt*, mitochondrion; *cp*, chloroplast. **(**B**)** Growth curve and culture images of *ALFA-mC-DRP5*$^{ESD::NbALFA\text{-}SKP1}$ cells cultivated asynchronously under continuous light with estradiol (0.1 µM) or vehicle (acetone). Estradiol was added at Day 0. Culture growth was monitored by measuring the optical density at 750 nm. Culture images were taken on Day 3. Growth curves represent the mean values of three independent cultures, and error bars indicate standard deviation. (C) Immunoblot analysis of synchronized cells during the third round of the 12-h light/12-h dark (LD) cycle. Estradiol or vehicle (acetone) was added 4 h before the onset of the third LD cycle (blue arrowhead). Synchronized cell division, including chloroplast division, occurred predominantly between 12 and 16 h. Anti-GFP and anti-SKP1 antibodies were used to detect ALFA-mC-DRP5B and NbALFA-SKP1, respectively. The CBB (Coomassie Brilliant Blue) -stained PVDF membrane served as a loading control. The predicted sizes of ALFA-mC-DRP5B and NbALFA-SKP1 were 136 and 33 kDa, respectively. (D) Differential interference contrast (DIC) and fluorescence microscopy of synchronized cultures. In the absence of estradiol, green fluorescence at the chloroplast division site indicated ALFA-mC-DRP5B localization (12 h), and magenta (chlorophyll) fluorescence marked the chloroplasts. In the presence of estradiol, ALFA-mC-DRP5B fluorescence was markedly reduced (double arrowheads), and chloroplast division was inhibited at 12 h. Arrows indicate cells undergoing irregular cytokinesis (16 and 24 h), and asterisks indicate dead, disorganized cells that had lost chloroplast fluorescence. Scale bar, 5 µm. (E) Frequency of cells undergoing cytokinesis with an undivided chloroplast (irregular cytokinesis) in synchronized culture. Values represent the mean of three independent cultures, and error bars indicate standard deviation. For each replicate, at least 150 cells were analyzed. (F) DAPI staining (DNA, blue) and chloroplast autofluorescence (Chl, magenta) showing a cell undergoing normal cytokinesis with divided chloroplasts (upper left) and a cell after cytokinesis (G_1, lower left), as well as irregular cytokinesis resulting from an undivided chloroplast (upper right). This irregular cytokinesis resulted in chloroplast-deficient cells (lower right). *n*, nucleus; *mtn*, mitochondrial nucleoid; *cpn*, chloroplast nucleoid. Scale bar, 2 µm.
